# Association Rate Constants of Ras-Effector Interactions Are Evolutionarily Conserved

**DOI:** 10.1371/journal.pcbi.1000245

**Published:** 2008-12-19

**Authors:** Christina Kiel, Dorothee Aydin, Luis Serrano

**Affiliations:** 1 EMBL-CRG Systems Biology Unit, Centre de Regulacio Genomica, Barcelona, Spain; 2 EMBL-European Molecular Biology Laboratory, Heidelberg, Germany; 3 ICREA Professor, EMBL-CRG Systems Biology Unit, Centre de Regulacio Genomica, Barcelona, Spain; University of Illinois at Urbana-Champaign, United States of America

## Abstract

Evolutionary conservation of protein interaction properties has been shown to be a valuable indication for functional importance. Here we use homology interface modeling of 10 Ras-effector complexes by selecting ortholog proteins from 12 organisms representing the major eukaryotic branches, except plants. We find that with increasing divergence time the sequence similarity decreases with respect to the human protein, but the affinities and association rate constants are conserved as predicted by the protein design algorithm, FoldX. In parallel we have done computer simulations on a minimal network based on Ras-effector interactions, and our results indicate that in the absence of negative feedback, changes in kinetics that result in similar binding constants have strong consequences on network behavior. This, together with the previous results, suggests an important biological role, not only for equilibrium binding constants but also for kinetics in signaling processes involving Ras-effector interactions. Our findings are important to take into consideration in system biology approaches and simulations of biological networks.

## Author Summary

Cellular signal transductions processes are based on protein interactions. Proteins can either associate transiently with each other or form stable complexes, and the strength of the interaction is described by the affinity (the affinity is the ratio between the rate of dissociation and association). Protein complexes with similar affinities can bind and dissociate with different rates, and these rates describe the kinetic properties of protein binding. These kinetic rates are important for signaling; however, to what extent individual changes in such rate constants are biologically important or whether the affinity is more crucial might be different in different signaling processes. In this study we analyze whether association rates are conserved during evolution, because evolutionary conservation of protein biochemical properties is usually a valuable indication of its importance. We analyzed the binding of Ras proteins to effector domains, which are central proteins in many signal transduction pathways, in different organisms. On the basis of homology modeling and energy calculations we find that association rates are conserved, although the sequence similarity decreases compared to the human protein. Our finding should encourage further analysis of the importance of kinetics for cellular signal transduction.

## Introduction

Protein-protein interactions are the central elements in all signal transduction processes. The life times of protein complexes as well as regulatory processes need to be tightly controlled for proper systems functioning. Affinities are used to characterize the strength of protein interactions and the affinities between proteins involved in signaling processes have been shown to correlate with the activities (output/response) in such signal transduction processes [1],[2]. In the majority of the cases, affinities between proteins and protein-ligands are determined using equilibrium binding methods, like isothermal titration calorimetry and fluorescence based methods, while rate constants of association and dissociation are only rarely determined. However, correlations of either association or dissociation rate constants with *in vivo* activity suggest that kinetic properties play a role in the cellular context [3]–[7]. As the affinity (K_d_) can be described as the ratio between the dissociation (k_off_) and association (k_on_) rate constants, different ratios of k_on_ and k_off_ values can give rise to similar affinities. Kinetic rate constants have been shown to be important for signal transduction, however to which extent kinetics influence signaling might depend on the actual network and network topology. We could speculate that fast k_on_ and k_off_ values could result in rapid activation and deactivation upon short pulses of a stimulus, while slow ones could filter noise and result in prolonged signaling. If this is true it might open new aspects of cellular signal transduction regulation and could probably lead to conceptually new strategies in drug design. It is likely that the answer will depend on the network topology: rate constants might be important in some signaling branches, in others not.

Evolutionary conservation of protein composition and biochemical properties is usually a valuable indication for the cellular importance of a specific protein complex. In this study we have selected the Ras-effector complex formation, in order to analyze whether kinetic rate constants are evolutionary conserved. Ras proteins belong to the Ras superfamily of small GTPases and they have key roles in various signal transduction pathways, like proliferation and differentiation [8]. They act as molecular switches by cycling between an active GTP-bound and an inactive GDP-bound state [9],[10]. Active Ras (Ras·GTP) can interact with effector molecules such as the Ser/Thr kinase Raf. The resulting Raf activation triggers the MAP kinase pathway, which leads to the transcription of target genes in the nucleus [11],[12]. Other Ras·GTP binding effector proteins that have been identified are the PI3-kinase, members of the RalGDS family, and AF6 [13]–[16]. Effector proteins bind to Ras·GTP via a common domain with a ubiquitin-like topology [17]–[22], and various structures of effector domains in complex with Ras proteins have revealed a similar binding mode that involves mainly two antiparallel ß-sheets of the RBD and Ras, respectively [23]–[29].

As Ras-effector protein interactions play a key role in cells, pathways involving Ras-effector interactions can be assumed to be at least partially conserved during evolution. In this study we analyzed whether the affinities and the association rate constants are conserved for 10 Ras-effector complexes in 12 different species, including worms, flies, fishes, and mammalian organisms. We used homology interface modeling and energy calculations, using FoldX 2.8 (http://foldx.crg.es/) [30],[31] in order to model Ras-effector interactions of proteins from different organisms. FoldX uses an algorithm based on the original work of Schreiber and co-workers to calculate relative changes in k_on_ which has been validated experimentally numerous times [32]. Homology modeling was performed in a similar way as done in a previous study, on a genome-wide level for all human Ras-effecter complexes [33],[34].

## Results

### Importance of Electrostatic Charge Complementarity for Ras-Effector Association Kinetics

Binding of effector proteins to Ras proteins is mediated via a domain with an ubiquitin-like topology [35]. Members of the ubiquitin domain superfamily are the RA, the RBD, the PI3Krbd, the UBQ and the B41/ERM domain families [36]. However, the binding of Ras to effector domains does not depend on the fold itself, but rather on certain amino acid residues on the surface that are crucial for binding.

**Figure 1:**
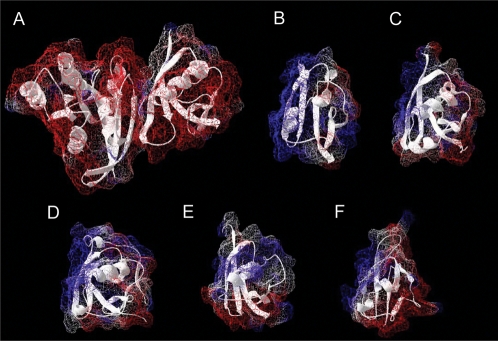
Electrostatic surface representation of Ras effector complex interfaces. (A) The Ras-RalGDS complex (pdb entry: 1lfd), and they single domains of (B) Rgl1 (pdb-entry: 1ef5), (C) Rgl2 (pdb-entry: 1rlf), (D) Grb7 (pdb-entry: 1wgr), (E) AF6-RA1 (pdb-entry: 1wxa), and (F) PLCeRA2 (pdb-entry: 2c5l) are shown.

An important observation found in Ras-effector complex structures is the high charge complementarity between the proteins of the complex, where Ras is mainly negatively charged and the effector RBDs are mainly positively charged [23]–[29]. Various studies have shown that a strong electrostatic surface complementarity in a protein complex enhances the association rate constant by forming of a low affinity encounter complex before the final high affinity complex is formed [37]–[42]. The complex formation itself is promoted by electrostatic steering which stabilizes the transition state by decreasing the energy barrier for association [42],[43]. In agreement with this concept of electrostatic steering and encounter complex formation, the association rate constants between Ras and effector domains were found to be fast (reviewed in [44]). Interestingly, the variance in binding energies when comparing different Ras-effector complexes is mainly the consequence of different association rate constants, while the dissociation rate constants are in a similar range [45]–[47]. For example, RafRBD is highly positively charged in its Ras binding region, and here the association rate constant was found to be very high in complex with the mainly negatively charged Ras proteins. In contrast, RalGDS has a mixed charged distribution (Figure 1A), and the k_on_ in complex with Ras is much lower. Interestingly, introducing positively charged residues at the edge of the interface of RalGDS can change binding kinetics and these RalGDS mutants were shown to bind “Raf-like” to Ras [43]. In Figure 1B–F we show the electrostatic surface potentials of several other RA/RB domains, which can bind to Ras (Rgl1, Rgl2, Grb7, AF6_RA1, PLCe_RA2) and for which structures have been solved, either by NMR or X-Ray, and we orient them similar as the RA domain of RalGDS in complex with Ras. In all cases the interface surface areas have a strong positively charged electrostatic potential, which suggests that association kinetics are important for these RA/RBD domains as well.

### FoldX Electrostatic Interactions and Association Rate Constants Correlate with Experimental Association Rate Constants in Ras/Raf and Ras/RalGDS Complexes

**Figure 2:**
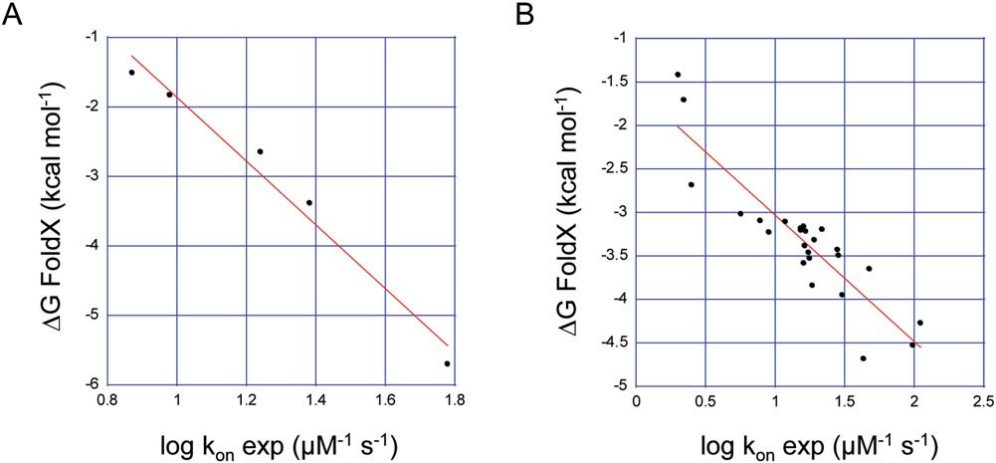
Calculation of association rate constants using FoldX and comparison with experimental asoociation rate constants. (A) Ras-Raf complex at different ionic strength (salt concentrations form 0 to 800 mM NaCl) (Table S1, [48]). The correlation coefficient is 0.987. (B) The Ras-RalGDS complex. Mutations within the RalGDS domain were introduced, and compared with experimental data (Table S1, [43]). The correlation coefficient is 0.892.

Although the algorithm developed by Schreiber and co-workers implemented in FoldX (http://foldx.crg.es/) [30],[31], has been validated experimentally on many different proteins, still it is a prediction method and as such needs some validation on the particular system under study. For this, we have selected the Ras-Raf complex and calculated k_on_ values (ΔG kon) at different salt concentrations, ranging from 0 to 800 mM NaCl (corresponding to an ionic strength of ∼50 to 850 mM in 50 mM Tris-buffer), and compared these results with experimental k_on_ values measured at different ionic strength using stopped-flow (Table S1; [48]). The experimental k_on_ values range from 7.4 to 60 µM^−1^ s^−1^ and an excellent correlation with calculated association rate constants was observed (R = 0.99) (Figure 2A). Further, we used FoldX in order to generate *in silico* a series of mutations of charged residue in RalGDS, located either in the binding site, or at the edge of the binding site, and we calculated binding energies as well as association rate constants using the Ras-Ral complex. When comparing these results with experiments [43], we find again a very good correlation between experimental and calculated k_on_ values (Figure 2B) (R = 0.89), with the slopes of the two correlations (ionic strength and mutants) been similar. This indicates that absolute values of association rate constants can be reliably calculated over a wide range for different ionic strengths and mutations of Ras-effector complexes.

### Ortholog Prediction of Ras and Selected Effector Proteins

**Figure 3:**
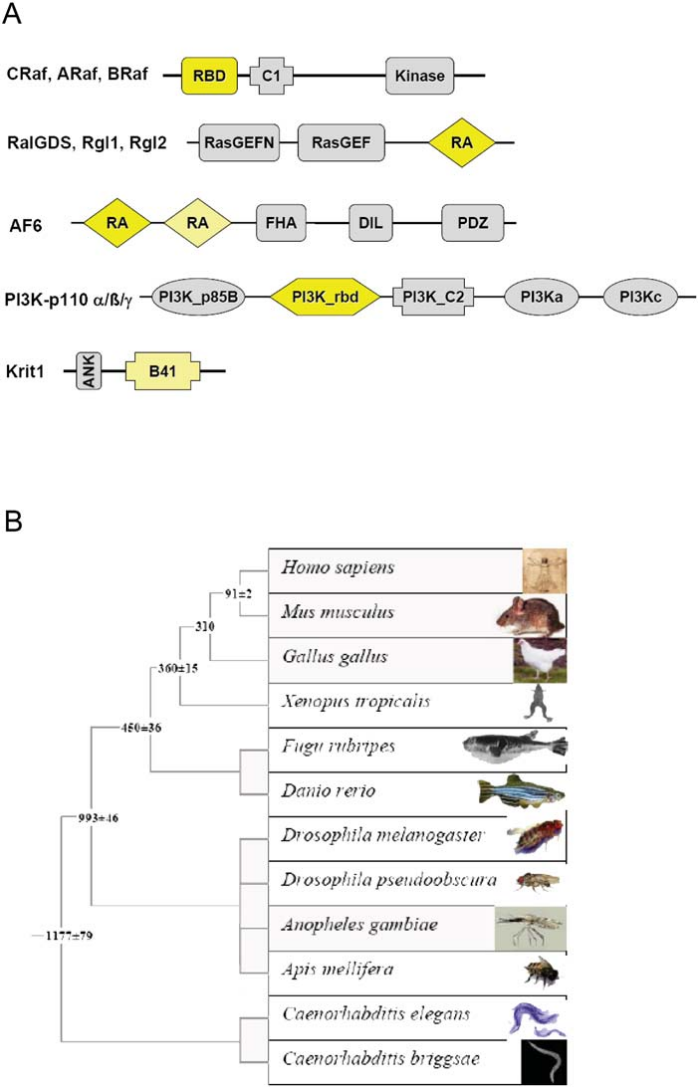
Selected effector domains and the phylogeny of used model organisms. (A) Domain architecture of selected effector proteins. The ubiquitin-like domains mediating binding to Ras (RA, RBD, PI3K_rbd, and B41) are shown in yellow, other domains in grey. The domain prediction was done using SMART [52],[53] using the respective sequence from *Homo sapiens*. (B) The relationship and divergence time (million of years ago (Mya)±standard error) of used model organisms are shown. The divergence times for the chordate – arthropod divergence and the one for divergence of nematodes from the lineage leading to chordates and arthropods were derived from Wang et al 1999 [61]. The others were taken from Hedges 2002 [62].

We have selected proteins containing RA, RBD, PI3Krbd, and B41 domains, similar as in our previous genome-wide Ras-effector homology interface modeling study [34] (Figure 3A), for which binding to Ras has been shown experimentally (Table 1). These include the different isoforms of the Raf kinases, RalGDS, and the related proteins, Rgl1, and Rgl2. Other Ras binding domains are the PI3K-p110 gamma subunit, and Krit. In the following we will often refer to members of the ubiquitin superfamily as UBDs, without differentiating between RA, RBD, PI3Krbd, or B41.

**Table 1:** Selected Ras binding domains, structural information, and template structures used for modeling.

Effector RBD	PDB Single Domain	PDB Complex with Ras Protein	Template Structure Used for Modeling
AF6 RA1	1wxa		1lfd
AF6 RA2			1lfd
Araf	1wxm		1gua
Craf	1rfa, 1rrb	1gua	1gua
Braf			1gua
Krit1			1lfd
PI3K p110 gamma		1he8	1he8
RalGDS	1lxd, 2rgf	1lfd	1lfd
Rgl1	1ef5		1lfd
Rgl2	1rlf		1lfd

In order to derive Ras and effector protein “interactions” from organisms representing the major eukaryotic branches, we have selected the following species (Figure 3B): *Homo sapiens* (*hs*) and *Mus musculus* (*mm*) were chosen for mammals, *Gallus gallus* (*gg*) for birds, *Xenopus tropicalis* (*xt*) for amphibians, *Fugu rubripes* (*fr*), and *Dario rerio* (*dr*) for fishes, *Drosophila melanogaster* (*dm*), *Drosophila pseudoobscura* (*dp*), *Anopheles gambiae* (*ag*) and *Apis mellifera* (*am*) for arthropods, and *Caenorhabditis elegans* (*ce*) and *Caenorhabditis briggsae* (*cb*) for nematodes. The orthologs were predicted by using the ENSEMBL (http://www.ensembl.org) [49] and the IMPARANOID databases (http://inparanoid.cgb.ki.se/) [50],[51]. Domains were predicted using SMART [52],[53] and the sequences were aligned automatically and by manual curation taking structural information into account [35] (Figure S1). Depending on the organism, between 22% and 78% of all human proteins orthologs were identified. When taking into account that certain proteins in lower organisms are orthologs of more than one human protein, e.g., RalGDS of *C. elegans* is also an ortholog of Rgl1 and Rgl2, the number of orthologs in different organisms ranges from 33% to 100%. The alignments of the UBDs of Ras effector proteins show a high similarity within orthologs and often also between different proteins of the same domain family. Furthermore, the similarity within the secondary structures of the RBD is higher than within the loops, indicating a conservation of the binding mode. The sequence identity of ortholog proteins (for detailed description see method) ranges between 100 and ∼20% (Table S2). However, in the majority of the cases the sequence identity decreases to ∼30/40%. The only exceptions are the different PI3kinase p110 isoforms, where a drastic drop in sequence identity is observed for the corresponding othologs/isoforms in *C. elegans*/*C. briggsae*.

The sequences of proteins that have a key role in cells are usually highly conserved among all organisms. In accordance with this, the sequences of Ras proteins were found to be nearly identical, especially in the effector binding region (Figure S2). The three Ras proteins, H-Ras, N-Ras and K-Ras could only be found in vertebrates, for arthropods and nematodes there is only one Ras protein which is most likely to be an ortholog of H-Ras. Due to the similarity in the effector binding region, only HRas was modeled (here termed as Ras).

### Homology Interface Modeling of Ortholog Ras-Effector Complexes

The first three secondary structure elements (β1, β2, and α1) of the ubiquitin-like domain determine the interaction surface towards Ras and they have the largest impact on binding energy of the complex [33]. In those cases in which a crystal structure of Ras in complex with a RBD domain was available we use the structure to model the ortholog sequences (Ras-Raf, Ras-Ral, Ras-PI3 Kinase, Ras-Byr). For the rest we used the templates modeled in our previous study [34] (Table S3) that were validated experimentally by pull-down experiments (for details see methods). Only those UB domains that could be reliably modeled were selected (e.g., no van der waals’ clashes above a fixed threshold of 2 kcal/mol). The species were then grouped into human (*hs*), mouse (*mm*), birds (*gg*), amphibians (*xt*), insects (*dm*, *dp*, *ag*, *am*) and nematodes (*ce*, *cb*). The mean of ΔG and ΔG_kon_ within each group was calculated and taken as value for the complete group. By grouping the different organisms, the problem of missing sequences can be solved for many proteins and mean values as well as standard deviation of ΔG and ΔG_kon_ can be calculated (the results do not change if we consider individual organisms, data not shown).

**Figure 4:**
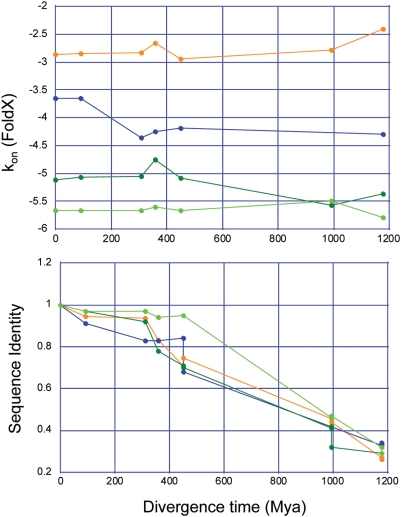
Homology interface modeling of ortholog Ras-effector complexes. Representative Results for predicted association rate constants. Association rate constants are conserved for Ras-effector complexes in different organisms with increasing divergence time, while the sequence identity is decreasing. Color code: AF6_RA2 (orange), RalGDS (dark blue), BRaf (light blue), CRaf (dark green), and AF6_RA1 (light green).

**Figure 5:**
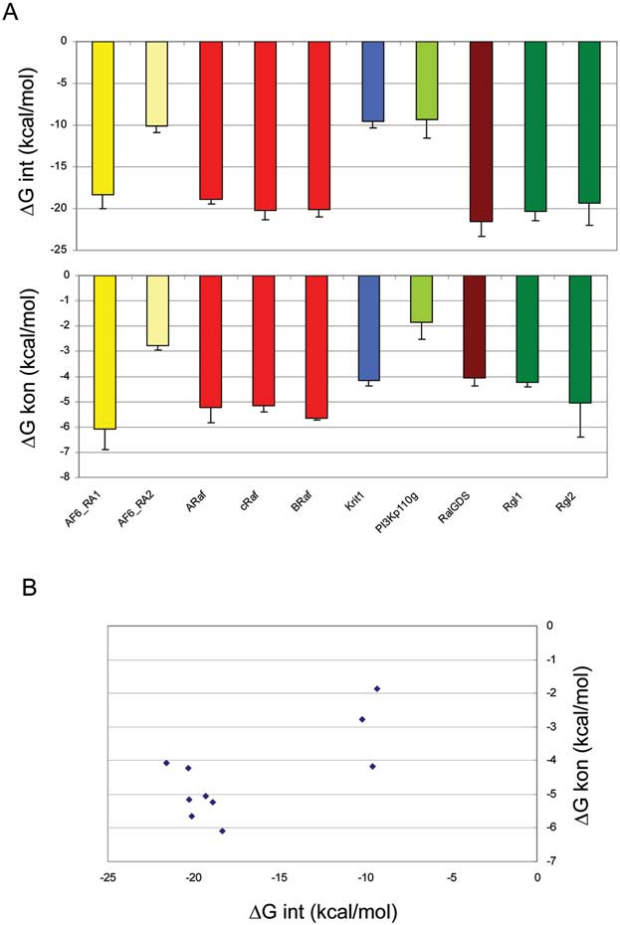
Mean values and standard deviation for calculated interaction energies (ΔGint) and association rate constants (ΔGkon). (A) Results from UBDs from the same protein or from the same protein family were shown in similar color. The results were taken from Table S5. (B) Correlation of calculated interaction energies with calculated association rate constants. The results were taken from Table S5, and the correlation coefficient is 0.71.

The results for all interaction energies (ΔGint FoldX) and contribution of association rate constants (ΔGkon FoldX) were plotted against the divergence time (Table S4 and Figure S3). While the sequence similarity decreases with increasing divergence time, the interaction energies as well as the association rate constants are conserved. A selection of representative results is shown in Figure 4. A comparison of the mean values for all interaction energies and k_on_ values calculated for a particular Ras-effector complex in different organisms shows that the standard deviations are in the majority of the complexes small (Table S5 and Figure 5A). Interestingly, the interaction energies correlate with the association rate constant contribution (Figure 5B) (R = 0.71). This indicates that also for the so far kinetically uncharacterized UBD domains, the changes in ΔG are mainly a consequence of changing k_on_. Thus, this could be underlying binding principle for the complete Ras-effector family.

In order to demonstrate that large changes in association rate constants would have been possible theoretically, we have selected the human Ras-RalGDS complex as an example for an *in silico* mutagenesis using FoldX. By either introducing positively or negatively charged residues at all positions at the surface of RalGDS, the FoldX-kon contribution could be increased from −3.65 kcal/mol to −7,6 kcal/mol or decreased to −0.47 kcal/mol, respectively (data not shown).

### Simulation of a Minimal Ras-Effector Network

**Figure 6:**
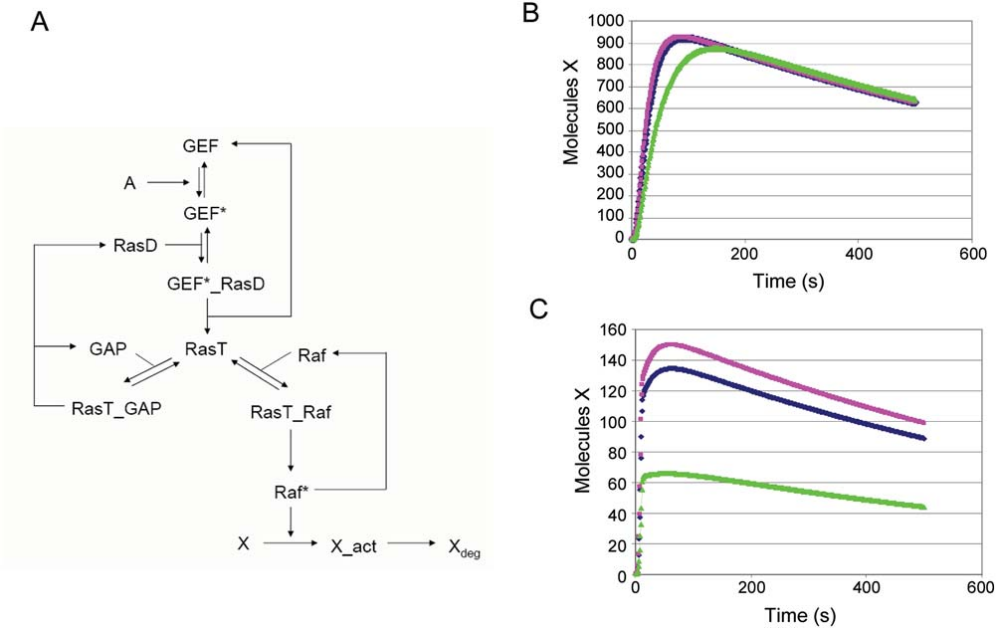
SmartCell Simulations of a minimal network within EGF signal transduction. (A) Schematic diagram of the reactions involved. For details see text and Table S5. Simulation of the network using the WT affinity of the Ras-Raf complex (blue line), 10 fold higher k_on_ and koff rates (pink line), or 10 fold lower k_on_ and k_off_ values (green line). Either a constant stimulus was applied for 500 seconds (B) or the system was activated for 10 seconds and then the stimulus was removed (C).

In order to analyze whether compensating changes in k_on_ and k_off_ can influence signal transduction, we used *in silico* simulations of a sub-network within the EGF signal transduction pathway. Activation of proteins following EGF stimulation is one of the most studied signaling systems, which involves the Ras-CRaf interaction as central elements, and numerous simulation models exist, which are able to correctly predict different aspects of EFG signaling found experimentally [54]–[59]. Based on these earlier models we have constructed a minimal network involving Ras and Raf kinase (Figure 6A and Table S6). This minimal model involves activation of GEF upon stimulation (A), which results in activation of Ras (RasT = RasGTP). Subsequent binding of Raf to RasT activates Raf (Raf_act), which in turn leads to activation of a downstream target (X). Negative regulation was introduced by the GAP catalyzed hydrolysis reaction of RasGTP to RasGDP (RasD). We simulated this network by first applying a constant stimulus of “A” for 500 seconds using the wild type k_on_ and k_off_ values for the Ras-Raf interaction. Then we simulated the network with either 10-fold higher k_on_ and k_off_ rate constants, or 10 fold lower k_on_ and k_off_ (Figure 6B). Minor changes are observed when following X over time (activation peak) for the simulation with 10-fold higher k_on_ and k_off_ compared to the wild type situation. Only the simulation of 10-fold lower k_on_ and k_off_ resulted in a slightly smaller activation peak. However, when simulating the network by applying a pulse of stimulation, of 10 s of “A” (and then removing the stimulus), large changes in the activation peak are observed, with a higher maximum for the simulation of 10-fold higher k_on_ and k_off_ values for the Ras-Raf interaction (Figure 6C). This shows, that under certain cellular conditions, like short pulse of activation, large changes in activation are expected for mutants with similar affinity, but changed and compensating effects on k_on_ and k_off_. Thus, kinetic properties can be crucial, and in the case of Ras-effector interactions, association kinetics will be important to result in sufficient activation, when the system is activated by applying a pulse.

## Discussion

The complex formation of Ras and effector proteins is driven by high association rate constants and only moderate dissociation rate constants [45]–[47]. Further, changes in affinity are mainly the consequence of changed association rate constants. Association rate constants can be influenced by mutating charged residues at the edge of the interface [32],[43]. If electrostatic interactions and association rate constants are important for the biological function of the cell, they should be conserved during the course of evolution. Using homology modeling and energy calculation covering a wide-range of sequences, and relating the output to the sequence conservation, we found that interaction energies as well as the electrostatic contributions and the association rate constants are conserved as well. While the sequence identity decreases with divergence time between the selected organisms, no trend could be found for the interaction energy and energies related to the electrostatics and k_on_, although theoretically it should be possible, when sampling the possible contributions of k_on_ at different amino acid positions (Figure S4).

Biologically, electrostatic interactions within Ras-effector complex interfaces could be functionally important, because they are the basis for the observed dynamic behavior, as observed in the case of Ras binding to the Raf kinase effector protein: The Ras-RafRBD complex formation is characterized by both high association and dissociation rate constants (k_on_ and k_off_), leading to affinities (K_d_ = k_off_/k_on_) in the range of 1 to 0.05 µM, under physiological conditions (this relatively low affinity seems to be functionally sufficient, since Ras is attached to the membrane via a lipid modification). The high k_on_ values provide the possibility to have a fast dissociation of the complex, while still having a reasonable tight binding complex (the lifetime of the complex between Ras and RalGDS, for example, is 0.1 s-1; see reference [47]). As Ras signaling depends very crucially on a strict control through regulating proteins like GAPs (GTPase activating proteins) and GEFs (guanine nucleotide exchange factors), this fast dissociation allows regulatory proteins to access and act.

We assume that electrostatics contributions and binding kinetics could be important in other Ras signaling pathways, since association rate constants were found to be conserved during evolution, as demonstrated in this study for 10 effector domains. Further *in vivo* analysis will be needed to prove this hypothesis. These experiments could be performed by designing mutant variants, which are expected to have similar affinities, but changed association and dissociation rate constants. These protein variants could be expressed in cells and the effect on signal transduction monitored, e.g., after different pulses of stimulation.

It is expected that the effect of changing rate constants depends also on the network topology (negative feedback, feed forward inhibition, etc). This knowledge will be important for systems biology and simulation approaches, in order to know, at which positions in the network affinities will be sufficient, while for other accurate rate constants will be crucial for correct prediction. Further, it could open conceptually new aspects in drug design.

## Methods

### Selected Species, Orthologs, and Domain Prediction

Proteins from the following species were used in order to get a good representation of all branches: *Homo sapiens* and *Mus musculus* (mammals), *Gallus gallus* (birds), *Xenopus tropicalis* (amphibians), *Fugu rubripes* and *Danio rerio* (fishes), *Drosophila melanogaster*, *Drosophila pseudoobscura*, *Anopheles gambiae* and *Apis mellifera* (arthropods), and *Caenorhabditis elegans* and *Caenorhabditis briggsae* (nematodes). Only two RA domain containing were retrieved from *Saccharomyces cerevisiae* (sc), because these proteins are involved in a different pathway. For each protein, the human ENSEMBL protein ID was retrieved from ENSEMBL (http://www.ensembl.org) [49]. The orthologs were predicted by using the ENSEMBL database for *Xenopus tropicalis*, or the INPARANOID database (http://www.inparanoid.cgb.ki.se) [50],[51]. ENSEMBL [49] classifies the prediction based on the BLAST results. Only those orthologs were chosen that were a unique best reciprocal hit in both directions. As the INPARANOID database [50],[51] provides more information about orthologs of members of protein families, e.g., PI3K p110, the prediction was preferentially used. The sequences retrieved from ENSEMBL or INPARANOID were analyzed using SMART (http://smart.embl-heidelberg.de) [52],[53], in order to determine the domain architecture of the protein and the domain sequences.

### Homology Modelling and Energy Calculations Using FoldX

For modeling of Ras-binding domains in complex with Ras proteins, we have taken the pdb-files of the following Ras effector complexes: Ras-RalGDS (pdb-entry: 1LFD), Ras-PI3Kinase (pdb-entry: 1HE8), and Raps-Raf (pdb-entry: 1GUA). Different template structures have been generated by deleting certain parts in the complex; the decision was mainly based on the alignment used to model the different binding domains. The ortholog sequences for one protein were aligned using standard automatic alignment tools, since sequence homology is high. However, the alignment of different effector domains from different families (RA, RBD, PI3Krbd, B41), was done based on manual curated structural-based sequence alignments as discussed in detail in a previous publication [35]. Basically two kinds of template structures have been generated (Table S3): a short version, where all secondary structure elements and loops (apart from ß1, ß1, ß2, a1) were deleted, as this is the part mainly contributing to the binding energy (similar as done in our previous study [33],[34]. In addition ‘long template’ structures have been generated. We could not model loop regions in those cases where the loops where not of the same length. For having a proline at the beginning of ß-strand 1 (position 26 in RalGDS, position 229 in PI3K, position 66 in Raf and position 81 in spByr2), we prepared special template structures by moving the backbone slightly, after introducing the proline at these positions (we checked that the proline was in acceptable dihedral angles and that the main chain CO group was still H-bonded to Ras). These template structures were then used to model the complex structures for AF6_RA2. The homology modelling was done as described before [33],[34]. The homology modeling was done based on the sequence alignment (Figure S1 and Figure S2), using different template structures using the design option in a new version of FoldX 2.8 [30],[31]. During this design procedure, FoldX is testing different rotamers and allows neighbor side chains to move. After reconstruction, all models have been passed through an additional optimization step by using the repair function of FoldX (detailed description in [33],[34]). Energy calculations of Ras-effector complexes have been done using FoldX as described before (http://fold-x.crg.es) [30],[31].

### Simulations of a Minimal Ras-Effector Network

A model was generated based on previous models of EGF signal transduction (see Table S6). Simulations were performed using the SmartCell software (http://www.smartcell-crg.es) [60] using ordinary differential equations.

## Supporting Information

Figure S1 Alignment of UB domains
(0.05 MB PDF)

Figure S2 Alignment of Ras proteins
(0.03 MB PDF)

Figure S3 Diagrams of DGint and DGkon FoldX values plotted against the divergence time
(0.07 MB PDF)

Figure S4 FoldX mutational scanning of RafRBD and RalGDS-RA. (A) Ras-RafRBD (pdb-entry 1GUA). (B) Ras-RalGDS (pdb entry 1LFD). Effect of all residues in Raf-RBD or RalGDS-RA on the contribution of DG kon as calculated by FoldX. Either positively charged residues were mutated to alanine (red) or negatively charged or neutral residues were mutated to lysine using FoldX and the difference compared to the WT DGkon was calculated and plotted for every amino acid position.
(0.03 MB PDF)

Table S1 Experimental and calculated association rate constants
(0.03 MB PDF)

Table S2 Divergence times and sequence identities for Ub domains
(0.02 MB PDF)

Table S3 Template structures used for homology modelling
(0.01 MB PDF)

Table S4 FoldX results for all homology models
(0.01 MB PDF)

Table S5 Mean values and STDEV for homology models for ortholog complexes
(0.01 MB PDF)

Table S6 Modelling parameters
(0.05 MB PDF)
