# Supplementary material for: Association Rate Constants of Ras-Effector Interactions Are Evolutionarily Conserved
Source: PLoS Comput Biol. 2008 Dec 19;4(12):e1000245. doi: 10.1371/journal.pcbi.1000245 (PMC2588540; doi:10.1371/journal.pcbi.1000245)
Supplement: Figure S1 — Alignment of UB domains (0.05 MB PDF) [file pcbi.1000245.s001.pdf]

```
Consensus   RA     pp  l+la          p*l l*p    pp* pcVlppll pc php       pca Lhph             -phh       cpPh h    a           pFhl+cc

hsRalGDS   2rgf    DCCIIRVSLDVD--NGNMYKSILVTS----QDKAPAVIRKAM-DKHNLEE----EEPEDYELLQILS-----------DDRKLKIP----ENANVFYAMNS---------TANYDFVLKKRT
hsRgl1             DTCIIRISVEDN--NGNMYKSIMLTS----QDKTPAVIQRAM-LKHNLDS----DPAEEYELVQVIS-----------EDKELVIP----DSANVFYAMNS---------QVNFDFILRKKNS
hsRgl2             DCRIIRVQMELG-EDGSVYKSILVTS----QDKAPSVISRVL-KKNNRDS----AVASEYELVQLLP-----------GERELTIP----ASANVFYAMDG---------ASHDFLLRQRRR
mmRalGDS           DCCIIRVSLDVD--NGNMYKSILVTS----QDKAPTVIRKAM-DKHNLDE----DEPEDYELVQIIS-----------EDHKLKIP----ENANVFYAMNS---------TANYDFLLKKRTF
mmRgl1             DTCIIRISVEDN--NGNMYKSIMLTS----QDKTPAVIQRAM-SKHNLESD---PAEEYELVQVIS-----------EDKELVIP----DSANVFYAMNS---------QVNFDFILRKKNS
mmRgl2             DCRIIRVQMELG-EDGSVYKSILVTS----QDKAPSVISRVL-KKNNRDSA----VASEFELVQLLP-----------GDRELTIP----HSANVFYAMDG---------ASHDFLLRQRRR
ggRalGDS           DCCIIRVSLAVD--NGNMYKSILVTS----QDKTPVVIRKAM-AKHNLDG----DRPEDYELVQIIS-----------EERELKIP----DNANVFYAMNS---------AANYDFVLKKRGF
ggRgl1             DTCIIRISVEDN--NGNMYKSIMLTS----QDKTPAVIQRAM-SKHNLESD---AAEEYELVQVIS-----------EDKELVIP----DSANVFYAMNS---------QVNFDFILRKKAP
frRalGDS           DCCIIRVSLDVE--NGNMYKSILVTS----QDKTPAVIRKAM-IKHNLER----EKTEDYELMQKIS-----------EDKELRIP----DNANVFYAMNS---------TANYDFVLKKRGL
frRgl1             DACIIRVSLEHG--NGNLYKSILLTN----QDKTPAVISRAM-AKHNLEVE----PDEGYELVQVIS-----------EERELVIP----DNANVFYAMNT---------SANFDFLLRVRGS
frRgl2             DMHIIRIRMDLQ--DGNLYRSILVTS----NDKTPTVISSAL-EKHNQDPK----HVSRYELIQLLP-----------EGKELIIP----ATGNVFYAMTT---------SSVDFLLRKKEG
drRalGDS           DACIIRVSLEQG--NGNLYKSIMLTS----QDKTPAVISRAM-TKHNLEG----EQAADYELVQVIS-----------EERELVIP----DNANVFYAMST---------SANFDFLLRLRGS
drRgl1             DACIIRVSLEQG--NGNLYKSIMLTS----QDKTPAVISRAM-TKHNLEGE----QAADYELVQVIS-----------EERELVIP----DNANVFYAMST---------SANFDFLLRLRGS
xtRalGDS           DCCIIRVSLDVD--NGNMYKSILVTS----QDKTPVVIRKAM-AKHNIDG----ERAEDYELVQIIS-----------EERELKIP----DNANVFYAMNS---------GANYDFVLKKRGF
xtRgl2             EHRIIRVRVEPA-EENSVYKSVRISS----QEKAPAVIDRIL-RKHHMTP------QGPQELIQLLP-----------EGKELVIP----EKANVFYAMS---------SASLDFLLRPRKT
ciRgl1             RFINVRIIRKGNEGGIPETKNMTLTI----NDRSHATIKQAT-KLFDIDD-----ESAKFSLSQILE-----------DNKELKIP----DTANVYYAMQS---------SCTNFNFALMEDGA
dmRgl1             DFYIIRVTYETD-5GIVLYKSIMLGN----NERTPQVIRNAM-LKLGLED-----DPDRFTLAQVL------------PDKELVMP----KNANVYYAVNT---------NYNLNFILRPRKE
dpRgl1             DFYIIRVTYETD-5GIVLYKSIMLGN----NERTPQVIRNAM-LKLGLED-----DPDRFTLAQVL------------PDKELVMP----KNANVYYAVNT---------NYNLNFILRPRKE
agRgl1             DFYIIKVTYETE-6GIVLYKSIMLAN----NERTPQVIRNAM-LKLGLEG-----DPDRYTLAQVL------------PDKELLLP----NNANVYYAVNT---------AYNLNFILRPKKD
amRgl1             DFYIIKVTMESD-5GVVLYKSIMLSN----NERTPQVIRNAM-LKLGIEG-----SPDQYTLAQVL------------PDREMLLP----NSANVYYAVNT---------AHNLNFILRPRRE
ceRalGDS           SFYLARVGLDDD-4DGANYKCIKIEN----GDRMPQLVARAL-EKHLIED-----DKNTYLLVQLL------------PRGGEFVLP----DNCNPFYAMAP------DPTSPMLNLLLRKRDG
cbRalGDS           SFYLARVGLDDD-4DGANYKCIKIEN----GDRMPQLIARAL-EKHLIDD-----EKNKYLLVQLL------------PRGGEFVLP----DNCNPFYAMAP------DPTSPMLNLMLRKRDC

hsAF6_RA1          FHGVMRFYFQDKAAGNFATKCIRVSS----TATTQDVIETLA-EKFRPDMRM--LSSPKYSLYEVHV----------GEERRLDIDE---KKPLVVQLNWNKDD-------REGRFVLKNEND
mmAF6_RA1          FHGVMRFYFQDKAAGNFATKCIRVSS----TATTQDVIETLA-EKFRPDMRM--LSSPKYSLYEVHVS---------- GERRLDID----EKPLVVQLNWNKDD-------REGRFVLKNEND
ggAF6_RA1          FHGVMRFYFQDKAAGNFATKCIRVSS----TATTQDVIETLA-EKFRPDMRM--LSSPKYSLYEVHVS----------GEERRLDVD----EKPLVVQLNWNKDD-------REGRFVLKNEND
frAF6_RA1          FHGVMRFYFQDRVAGNFATKCIRVSS----TATTQDVIETLA-EKFRPDMRM--LSSPKYSLYEVHVS----------GEERQLDLD----EKPLVVQLNWNKDD-------REGRFVLKNEND
drAF6_RA1          FHGVMRFYFQDRVAGNFATKCIRVSS----TATTQDVIETLA-EKFRPDMRM--LSSPKYSLYEVHVS----------GEERKLDLD----EKPLVVQLNWNKDD-------REGRFVLKNEND
xtAF6_RA1          FHGVMRFYFQDKAAGNFATKCIRVSS----TATTQDVIETLA-EKFRPDMRM--LSSPRYSLYEVHVS----------GEERKLDFE----EKPLVVQLNWNKDD-------REGRFVLKNEND
dmAF6_RA1          FHGVMRFYFQD-AGQKVATKCIRVAS----DATVTDVIDTLI-EKFRPDMRM--LSVPNYALYEVHAN----------GEERRLNAD----EKPLLVQLNWHIDD-------REGRFLLKNIDQ
dpAF6_RA1          FHGVMRFYFQD-AGQKVATKCIRVAS----DATVTDVIDTLI-EKFRPDMRM--LSVPNYALYEVHAN----------GEERRLNAD----EKPLLVQLNWHIDD-------REGRFLLKNIDQ
agAF6_RA1          FHGVMRFYFQD-EGQKVATKCIRVAS----DATVSDVIETLI-EKFRPDMRM--LSLPNYALYVVHAN----------GEERKLNPD----EKPLLVQLNWHNDD-------REGRFLLKNCAQ
ceAF6_RA1          VEGVMRFYFQD-GGEKVLTKCVRVSS----TATTRAVVDALS-EKFLPDLKM--LSNDTYSLWEVHEN----------GEERKLLDE----EKPLVVQLQWHKDD-------REGRFLLKKDAA
cbAF6_RA1          VEGVMRFYFQD-GGEKVLTKCVRVSS----TATTRAVVDALS-EKFLPDLKM--LSNDTYSLWEVHEN----------GEERRLGEE----EKPLVVQLQWHKDD-------REGRFLLKKDKS

hsAF6_RA2          SGGTLRIYADSLKP-NIPYKTILLST----TDPADFAVAEAL-EKYGLEKE----NPKDYCIARVMLPPGAQHSDEKGAKEEILLDDD---ECPLQIFREWPSDK-------GILVFQLKRRPP
mmAF6_RA2          SGGTLRIYADSLKP-NIPYKTILLST----TDTADFAVAESL-EKYGLEKE----NPKDYCIARVMLPPGAQHSDERGAKEIILDDD----ECPLQIFREWPSDK-------GILVFQLKRRPP
ggAF6_RA2          SGGTLRIYADSLKP-NIPYKTILLST----TDTADFAVIEAL-EKYGLEKE----NPKDYCIARVILPPGAQHSDDKGAKETILDDD----ECPLQIFREWPSDK-------GILVFQLKRRPP
frAF6_RA2          SGGTLRIYADSLKP-NIPYKTILLST----RDTADFAVVEAL-EKYGLEKE----NPREYCIARQDDKX---------GKDVILDDN----ECPLXIFRDWPADR-------GALVFQLKKRPP
drAF6_RA2          SGGTLRIYADSLKP-NIPYKTILLST----TDMADFAVAEAL-EKYGLEKE----NPRDYCIAQVIHA------NDKAVKETILDDT----DCPLQILRDWTSDK-------EALVFQLKKRPP
xtAF6_RA2          SGGTLRIYADSLKP-NIPYKTILLST----NDTADFAVIEAL-EKYGMEKE----NPKEYCIAKVVLPPGAQHSD-KGMKEFILDDE----ECPLHIFRDWPSEK-------GELVFQLKRRPT
dmAF6_RA2          TGGTLKIYGESLCQ-DVPYKTLLLSI----RDCAQAVVREML-TKYGLEKA----DPLHYCLVQVNSD----------GTEYILDDD----ECPLSILMNHPTSR-------GSIMFHVRRRPA
dpAF6_RA2          TGGTLKIYGESLCQ-DVPYKTLLLSI----RDCAQAVVREML-TKYGLEKA----DPLHYCLVQVNSD----------GTEYILDDD----ECPLSILMNHPTSR-------GSIMFHVRRRPA
agAF6_RA2          TGGTLKIYGESLCR-DVPYKTLLLSI----RDCAQAVVREML-SKYGMDKV----DPLHYCLVQVNSD----------GSEYILDDD----ECPLSILMNHPTSR-------GSIMFHVRRRPA
ceAF6_RA2          FGGSLKVYGGEIVP-TRPYVSILAEI----NENADRILGAAL-EKYGLEHS-----KDDFILVEVSNDDDRKSMSDLREIDGRPIPPT---ECPLFEMTARSGNGEN-----GFDSFLAIKRKPH
cbAF6_RA2          FGGSLKVYGGEIVP-TRPYVSILAEI----NETADRILIAAL-EKYGLEQS-----KDDFILVEVSNDDERKSMSDLRDVDGRPIPPT---ESPLFEMTARGNGEH------GFDSFLAIKRK

hsKrit1          NKPYEKVRIYRM-----DGSYRSVELKHGNN------TTVQQIM-EGMRL--------SQETQQ-YFTIWICSEN----(35)-QLFLRRDVRLPLEVE--------------KQIE-DPLAILILF
mmKrit1          NKPYEKVRIYRM-----DGSYRSVELKHGNN------TTAQQIM-EGMRL--------SQETQR-YFTIWICSEN----(35)-QLFLRRDVGLPLEVE--------------KKIE-DPLAILILF
ggKrit1          NKPYEKARIYRM-----DGSYRSVELKHGNN------TTVQQIM-EGMRL--------SQETQQ-YFTIWICSEN----(35)-QLFLRRDVRLPLEIE--------------KKIE-DPLAILILF
frKrit1          SRPYEKVRIYRM-----DGSYRSVELKHGNN------TTVQQIM-EGMRL--------TQDTQQ-YFTIWICSEN----(35)-QLFLRRDVRLPLEIE--------------KKVE-DPLAILILF
xtKrit1          NKPYEKVRIYRM-----DGSYRSVELKHGNN------NTVQQIM-EGMRL--------SQETQQ-YFTIWICSEN----(35)-QLFLRRDVKLPLDVE--------------KKIE-EPLAILILF
```

```
Consensus            hpl LP       p  hV h+ G     plc- Lp hl c +GLp         p h Va h p             cc l hpp    L                     pcl l-

hscRaf      1c1y  SNTIRVFLP-----NKQRTVVNVRNGM----SLHDCLMKAL-KVRGLQ--------PECCAVFRLLHEH--------KGKKARLDWNTDAASLIG-------------------EELQVDFL
hsARaf             GTVKVYLP-----NKQRTVVTVRDGM----SVYDSLDKAL-KVRGLN--------QDCCVVYRLIK-----------GRKTVTAWDTAIAPLDG-------------------EELIVEVL
hsBRaf             PIVRVFLP-----NKQRTVVPARCGV----TVRDSLKKAL-MMRGLI--------PECCAVYRIQD-----------GEKKPIGWDTDISWLTG-------------------EELHVEVL
mmcRaf             NTIRVFLP-----NKQRTVVNVRNGM----SLHDCLMKAL-KVRGLQ--------PECCAVFRLLQEH--------KGKKARLDWNTDAASLIG-------------------EELQVDFL
mmARaf             GTVKVYLP-----NKQRTVVTVREGM----SVYDSLDKAL-KVRGLN--------QDCCVVYRLIKG-----------RKTVTAWDTAIAPLDG-------------------EELIVEVL
mmBRaf             PIVRVFLP-----NKQRTVVPARCGV----TVRDSLKKAL-MMRGLI--------PECCAVYRIQDG-----------EKKPIGWDTDISWLTG-------------------EELHVEVL
ggcRaf             NTIRVFLP-----NKQRTVVNVRNGM----TLHDCLMKAL-KVRGLQ--------PECCAVFRLVTEP--------KGKKVRLDWNTDAASLIG-------------------EELQVDFL
ggBRaf             PIVRVFLP-----NKQRTVVPARCGV----TVRDSLKKAL-MMRGLI--------PECCAVYRIQDG-----------EKKPIGWDTDISWLTG-------------------EELHVEVL
frcRaf             STIRVYLP-----NQQRTVVNVRPGM----TLHSCLIKAL-KVRGLQ--------PQCCAVFRLHPGQ--------SSKKLRMDWNTDSTSLIG-------------------QELLVEVL
frARaf             GTIRVYLP-----NKQRTVVNVRQGQ----TVHESLDKAL-KVRGLN--------QDCCAVFRLLEG-----------RKRLTDWDTDITPLVG-------------------EELLVEVL
drcRaf             RTIRVFLP-----NQQRTVVNVRPGM----TLHSCLIKAL-KVRGLQ--------PECCAVFSLHPGQ--------RSKKSRMEWSTDSTSLIG-------------------EELLVEVL
drARaf             GTIRVYLP-----NKQRTVVNVRPGQ----TVYDSLDKAL-KVRGLS--------QDCCAVFRLLEG-----------RKRLTEWDTDITPLVG-------------------EELLVEVL
drBRaf             PIVRVFLP-----NKQRTVVPARCGM----TVRDSLKKAL-MMRGLI--------PECCAVYRVQDG-----------EKKPIGWDTDISWLTG-------------------EELHVEVL
xtcRaf             SPMRVYLP-----NKQRTVVNVRSGM----SLHDCLMKAL-KVRGLQ--------PECCAVFRLIQDP---------KGKLRLDWNTDAMSLVG-------------------AELQVDFL
xtBRaf             PIVRVFLP-----NKQRTVVPARSGV----TVRDSLKKAL-MMRGLI--------PECCAVYRVQDG-----------EKKPIGWDTDISWITG-------------------EELHVEVL
ciBRaf             GVIRIYLP-----NGKTTVGAIPGKR----LKEVLLKSAR-LKSLRD-------KLDQCSVYRFG-------------TDDHVDWNSDSGILAG-------------------VQLELR--
dmRaf              ILLRAHLP-----NQQRTSVEVISGV----RLCDALMKAL-KLRQLT--------PDMCEVSTTHS------------GRHIIPWHTDIGTLHV-------------------EEIFVRLL
dpRaf              ILLRAHLP-----NQQRTSVEVVAGV----RLCDALMKAL-KLRQLT--------PDMCEVSTSHS------------GRHIIPWNTDIGTLHV-------------------EEIFVRLL
agRaf              MLLRAFLP-----NQQRTSVQVIPGM----RLKDALAKAL-KRRNLT--------CEFCEVTAGNS-------------NYPIPWETDVSALNC-------------------DEVFVRIL
amBRaf             SLLRAYLP-----NQQRTSVQVREGL----SLRDALAKAM-KLRNLT--------TEMCVVYILGAD----------NSRYLTSWDTDISLLDC-------------------DEISVEIL
ceRaf              KMIMVHLP-----FDQHSRVEVRPGE----TARDAISKLL-KKRNIT--------PQLCHVNASSDPK-------QESIELSLTMEEIASRLPG-------------------NELWVHSE
cbRaf              KMIMVHLP-----FDQHSRVEVRPGE----TARDAISKLL-KKRNIT--------PQLCQVNTSPDSK-------TEAIDLSKTMEEIALHLPD-------------------NELWVHSE

Consensus           pl l l pp     ppp  *lphp pp    *   lhpphh pK     *  pp       calL+VCG          pcEalh pp LhpapalppClKp             cl Lhp
hsPI3K-p110g      NNCIFIKIHRS------TTSQTIKVSPDD----TPGAILQSFF-TKMAKKKSLMDIPESQSEQDFVLRVCG----------RDEYLVGETPIKNFQWVRHCLKNGEE---------IHVVLDTPPD
hsPI3K-p110b      GGKLIVAVHFE----NCQDVFSFQVSPNM----NPIKVNELAI-QK---RLTIHGKEDEVSPYDYVLQVSG----------RVEYVFGDHPLIQFQYIRNCVMNRAL---------PHFILVECCK
hsPI3K-p110a      RGQIIVVIWVIVSPNNDKQKYTLKINHDC----VPEQVIAEAI-RKK--TRSMLLSSEQL(9)KYILKVCG----------CDEYFLEKYPLSQYKYIRSCIMLGRM---------PNLMLMAKES
hsPI3K-p110d      ALLVNVKFEGS------EESFTFQVSTKD----VPLALMACAL-RKK---ATVFRQPLVEQPEDYTLQVNG----------RHEYLYGSYPLCQFQVQKPRAKPP(7)--------SSVSLWSLEQ
mmPI3K-p110g      NNCIFIVIHRG------TTSQTIKVSADD----TPGTILQSFF-TKMAKKKSLMNISESQSEQDFVLRVCG----------RDEYLVGETPLKNFQWVRQCLKNGDE---------IHLVLDTPPD
mmPI3K-p110b      GGKLVVAVHFE----NSQDVFSFQVSPNL----NPIKINELAI-QK---RLTIRGKEDEASPCDYVLQVSG----------RVEYVFGDHPLIQFQYIRNCVMNRTL---------PHFILVECCK
mmPI3K-p110a      KGQIIVVIWVIVSPNNDKQKYTLKINHDC----VPEQVIAEAI-RKK--TRSMLLSSEQL(9)KYILKVCG----------CDEYFLEKYPLSQYKYIRSCIMLGRM---------PNLMLMAKES
mmPI3K-p110d      ALLVNVKFEGS------EESFTFQVSTKD----MPLALMACAL-RKK---ATVFRQPLVEQPEEYALQVNG----------RHEYLYGNYPLCHFQYICSCLHSGLT---------PHLTMVHSSS
ggPI3K-p110g      NNNIFIIIHRG------TTSQKIKVSIDD----TPDMILHSFF-TKMAKKKSLMDIPEDHSELDFVLRICG----------RDEYITGDTPIKDFHWIRQCLKNGEE---------IHLVLDNPPD
ggPI3K-p110a      KGQIIVVIWVIVSPNNDKQKYTLKINHDC----VPEQVIAEAI-RKK--TRSMLLSSEQL(9)KYILKVCG----------CDEYLLEKYPLSQYKYIRSCIMLGRM---------PNLMLMAKES
ggPI3K-p110d      NIFVNVKFQSG------GESFTFQISPNE----FPITLMSYAV-KKQ---ATVFRHETMENPEDYTLQVNG----------KYEYLYGNYPLYQFQVNSIANPPKMA---------VKPPPLPK
frPI3K-p110g      NGSILVVIHVS------TVSQTIKVSIGD----SPAQVLTSFF-AKTSNKRVLLGIPENLSETDFVLRVCG----------REEYLYGDKPLQNFNWVRQCLKNGEE---------IHLVLETPPN
frPI3K-p110b      PADLKIIIHFD----QSLDSASLKMASTC----TPRDLITQAV-RK---WLTTHGPEEDALRGQYVLRVSH----------CLEFLCGDHPLIQYKYIRTCMQAKEP---------PHVTLVHNNT
frPI3K-p110a      KGQIIVVIWVIVSPTNDKQKYTLKINHDY----VPEQVIAEAI-RKK--TRSMLLSQEQL(9)KYILKVCG----------CDEYLLEKYPLSQYKYVRSCIMLGRM---------PNLMLMAKDS
frPI3K-p110d      NTKIFINVKFE----ASDESFMLQQDPQD----LPAALMRAAL-KKK---ATVFRSVRQE-PEDYTLQVNG----------RWEYIYGRHPLSQFKVSGHEKLPP------------PLPLKKQNT
drPI3K-p110g      NNHILMVIHKE------TTSQTIKVSIDD----TPVQVLQSFF-AKITNKRALLGISEDVSESDFVLRVCG----------REEYLYGNNAIKDFHWIRQCLKNGEE---------IHLVLEHPPN
drPI3K-p110b/d    EGIIKITMHYD----QSQDTASLKVSLNC----TVSEVMDHAL-KK---WQTTHGPEE(4)RSNYVLRVSD----------RLEFLFEDYPLIQYKYIRACLSTGEN---------PHLTVVHFDI
drPI3K-p110a      KGQIIVVIWVIVSPNNDKQKYTLKINHDC----VPEQVIAEAI-RKK--TRSMLLSAEQL(9)KYILKVCG----------CDEYLLEKYPISQYKYVRSCIMLGRL---------PNLMLMSKDS
xtPI3K-p110g      SNSIFIIIHKG------MTSHTIRVSIDD----TPDVILHSFF-TKMAKKKSLLDISEENSELDFVLRVCG----------RDEYIIGNAPIKDFHWIRQCIKNGQE---------IHLVLEKPPD
xtPI3K-p110b      GGNIVVAIHFE----NCQDVFSFQVSPNM----VPVKVNELAI-RK---RLTIHGREA(4)PEDFVLQVNG----------RLEFVFGDHPLIQFQYIRNCVMNKAL---------PHLTLVECCN
xtPI3K-p110a      KGQIIVVIWVIVSPNNEKQKYSLKINHDC----VPEHVIAEAI-RKK--TRSMLLSSEQL(9)KYILKVCG----------CDEYLLEKYPLSQYKYIRSCIMLGRM---------PNLMLMAKDS
ciPI3K-p110g      TITVCIYGPS-------KSSTTMSVGVYD----PPSKIMEDFF-ES(4)RKIAFGIPEEAKAPDYVLKVCG----------MCDYIWGPHEIMDFVYIRHCVARRRE---------PQLALCVPPN
ciPI3K-p110a      NTNIVIRVWVVQEN-SFQTHASVCVPCDT----TPAMIISEVV-KKK-4NNSQSNSTKSIN-EHYVLKVCG----------LNEYLEGNFPICQYKYIRQCVNRDEL---------PDVMLESVKQ
dmPI3K-p110b/d    TRTFLIVVKNE----NDQSTFTLSVNEQD----TPFSLTESTL-QK--MNRSQMKMND--RTSDYILKVSG----------RDEYLLGDYPLIQFLYIQEMLSDSAV---------PNVVLQSVYR
dpPI3K-p110b/d    TSAFLIVVKNE----NDQSTFTLSVMEKD----TPFSLIESTL-QK--MNRSQMKMND--RAGDYILKVSG----------RDEYLFGDHPLIQFLYIQEALSDSAV---------PNVVLQSVIR
agPI3K-p110b/d    LTNGDFCVVAI----STDMQLTVKVPCRA----TPDEVLKRIL-KK--QNPSKARNEN--SSTEYILKVCG----------REEYIYGAHPMISFQYVQDCLSRDET---------PTFVPRLVRT
amPI3K-p110b/d    DGNIVLVTKFE----NTETAFTFQISHST----TPYQLLVSIL-NK--RANTLMSK---GHPNDFTLKVCG----------QEEYLIGDYPLIQFSYIQDCVAKDIT---------PTLVTLSRDS
cePI3K-p110       AAKLSYQMFWRK(7)VCEKMMKIQIEFNPNE--TPKSLLHTFL-YEM--RKLDVYDTDDPADEGWFLQLAG---------RTTFVTNPDVKLTSYDGVRSELESYRC---------PGFVVRRQS
cbPI3K-p110       AAKLDYQLFWTK(7)VWEKMYKITIDFDPEF--NPQSLMRMFV-KEL--QCMNLFDPEDPPDEEWILQLAG---------RTSFVTRPEISLVSYDGIRSELESYRC---------PGFVVRRKSL
```
