# Supplementary material for: Association Rate Constants of Ras-Effector Interactions Are Evolutionarily Conserved
Source: PLoS Comput Biol. 2008 Dec 19;4(12):e1000245. doi: 10.1371/journal.pcbi.1000245 (PMC2588540; doi:10.1371/journal.pcbi.1000245)
Supplement: Figure S2 — Alignment of Ras proteins (0.03 MB PDF) [file pcbi.1000245.s002.pdf]

```
                            hhhhhhhhhh                hhhhhhhhh  sssssssss                  hhhhhhhhh  ssssssss   hhhhhhh
Consensus Ras  ppYKlVVlG GGVGKSALTlQFlQ p--Fl-cYDPTI EDSYRKQhhlD  c  hLDILDTAGQEEa          AMRcQYMR GpGFllVYSlT +pSFcElp

hsHRas        MTEYKLVVVGAGGVGKSALTIQLIQNH--FVDEYDPTI-EDSYRKQVVID-GETCLLDILDTAGQEEYS---------AMRDQYMRTGEGFLCVFAINNTKSFEDIH
hsNRas        MTEYKLVVVGAGGVGKSALTIQLIQNH--FVDEYDPTI-EDSYRKQVVID-GETCLLDILDTAGQEEYS---------AMRDQYMRTGEGFLCVFAINNSKSFADIN
hsKRas        MTEYKLVVVGAGGVGKSALTIQLIQNH--FVDEYDPTI-EDSYRKQVVID-GETCLLDILDTAGQEEYS---------AMRDQYMRTGEGFLCVFAINNTKSFEDIH
mmHRas        MTEYKLVVVGAGGVGKSALTIQLIQNH--FVDEYDPTI-EDSYRKQVVID-GETCLLDILDTAGQEEYS---------AMRDQYMRTGEGFLCVFAINNTKSFEDIH
mmNRas        MTEYKLVVVGAGGVGKSALTIQLIQNH--FVDEYDPTI-EDSYRKQVVID-GETCLLDILDTAGQEEYS---------AMRDQYMRTGEGFLCVFAINNSKSFADIN
mmKRas        MTEYKLVVVGAGGVGKSALTIQLIQNH--FVDEYDPTI-EDSYRKQVVID-GETCLLDILDTAGQEEYS---------AMRDQYMRTGEGFLCVFAINNTKSFEDIH
ggHRas        MTEYKLVVVGAGGVGKSALTIQLIQNH--FVDEYDPTI-EDSYRKQVVID-GETCLLDILDTAGQEEYS---------AMRDQYMRTGEGFLCVFAINNTKSFEDIH
ggNRas        MTEYKLVVVGAGGVGKSALTIQLIQNH--FVDEYDPTI-EDSYRKQVVID-GETCLLDILDTAGQEEYS---------AMRDQYMRTGEGFLCVFAINNSKSFADIN
ggKRas        MTEYKLVVVGAGGVGKSALTIQLIQNH--FVDEYDPTI-EDSYRKQVVID-GETCLLDILDTAGQEEYS---------AMRDQYMRTGEGFLCVFAINNTKSFEDIH
frHRas        MTEYKLVVVGAGGVGKSALTIQLIQNH--FVDEYDPTI-EDSYRKQVVID-GETCLLDILDTAGQEEYS---------AMRDQYMRTGEGFLCVFAINNTKSFEDIH
frKRas        MTEYKLVVVGAGGVGKSALTIQLIQNH--FVDEYDPTI-EDSYRKQVVID-GETCLLDILDTAGQEEYS---------AMRDQYMRTGEGFLCVFAINNTKSFEDIH
drHRas        MTEYKLVVVGAGGVGKSALTIQLIQNH--FVDEYDPTI-EDSYRKQVVID-GETCLLDILDTAGQEEYS---------AMRDQYMRTGEGFLCVFAINNTKSFEDIH
xtHRas        MTEYKLVVVGAGGVGKSALTIQLIQNH--FVDEYDPTI-EDSYRKQVVID-GETCLLDILDTAGQEEYS---------AMRDQYMRTGEGFLCVFAINNTKSFEDIH
xtKRas        MTEYKLVVVGAGGVGKSALTIQLIQNH--FVDEYDPTI-EDSYRKQVVID-GETCLLDILDTAGQEEYS---------AMRDQYMRTGEGFLCVFAINNTKSFEDIH
xtNRas        MTEYKLVVVGAGGVGKSALTIQLIQNH--FVDEYDPTI-EDSYRKQVVID-GETCLLDILDTAGQEEYS---------AMRDQYMRTGEGFLCVFAINNSKSFADIN
dmRas         MTEYKLVVVGAGGVGKSALTIQLIQNH--FVDEYDPTI-EDSYRKQVVID-GETCLLDILDTAGQEEYS---------AMRDQYMRTGEGFLLVFAVNSAKSFEDIG
dpRas         MTEYKLVVVGAGGVGKSALTIQLIQNH--FVDEYDPTI-EDSYRKQVVID-GETCLLDILDTAGQEEYS---------AMRDQYMRTGEGFLLVFAVNSAKSFEDIG
agRas         MTEYKLVVVGAGGVGKSALTIQLIQNH--FVDEYDPTI-EDSYRKQVVID-GETCLLDILDTAGQEEYS---------AMRDQYMRTGEGFLLVFAVNSAKSFEDIG
amRas         MTEYKLVVVGAGGVGKSALTIQLIQNH--FVDEYDPTI-EDSYRKQVIID-GETCLLDILDTAGQEEYS---------AMRDQYMRTGEGFLLVFAVNSAKSFEDIG
ceRas         MTEYKLVVVGDGGVGKSALTIQLIQNH--FVEEYDPTI-EDSYRKQVVID-GETCLLDILDTAGQEEYS---------AMRDQYMRTGEGFLLVFAVNEAKSFENVA
cbRas         MTEYKLVVVGDGGVGKSALTIQLIQNH--FVEEYDPTI-EDSYRKQVVID-GETCLLDILDTAGQEEYS---------AMRDQYMRTGEGFLLVFAVNEAKSFENVA
```
