# Supplementary figures and images for: Association Rate Constants of Ras-Effector Interactions Are Evolutionarily Conserved
Source: PLoS Comput Biol. 2008 Dec 19;4(12):e1000245. doi: 10.1371/journal.pcbi.1000245 (PMC2588540; doi:10.1371/journal.pcbi.1000245)

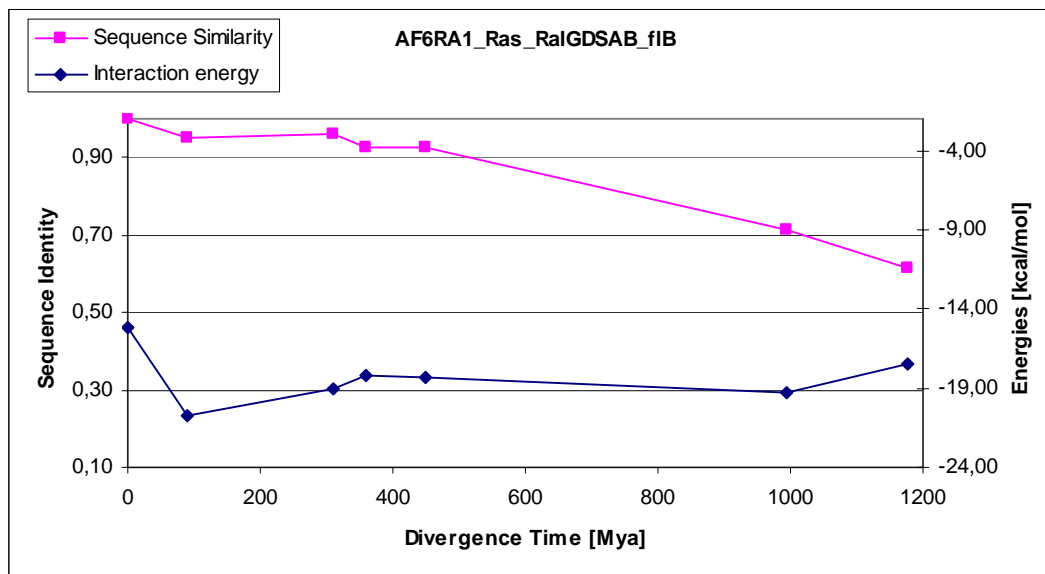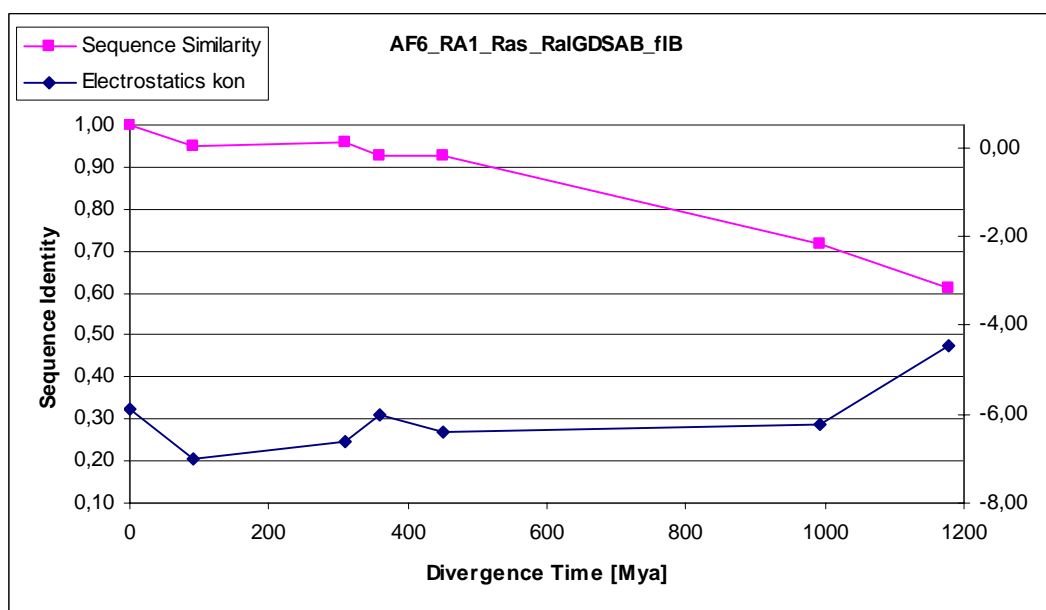

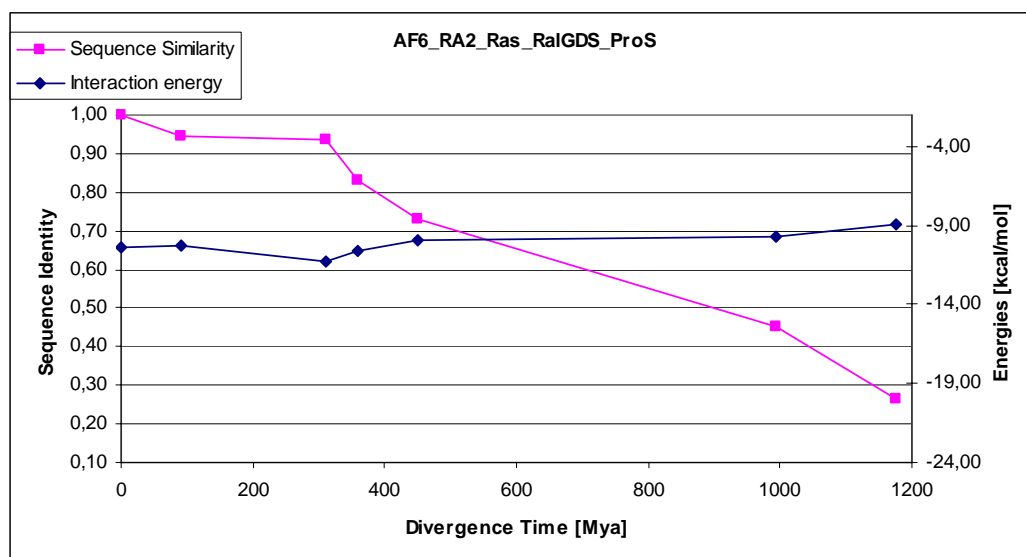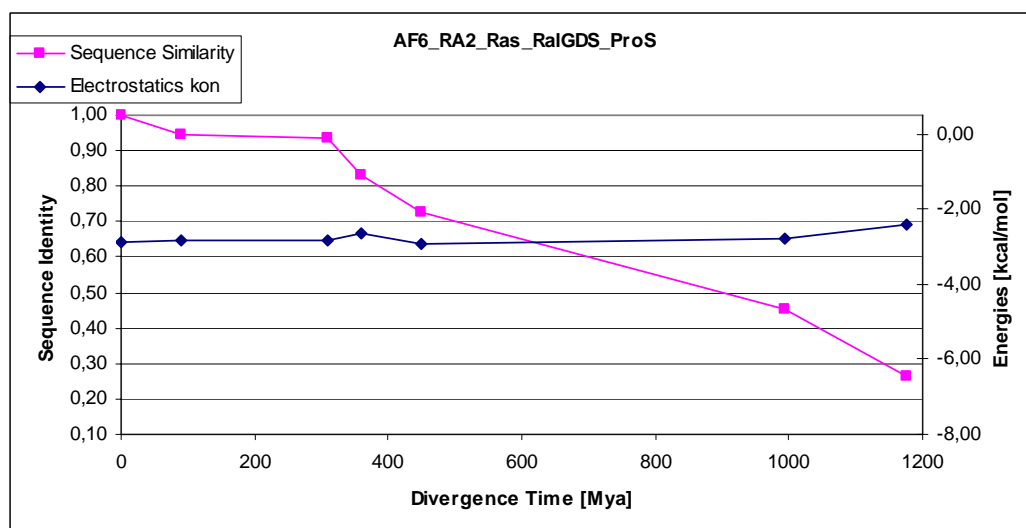

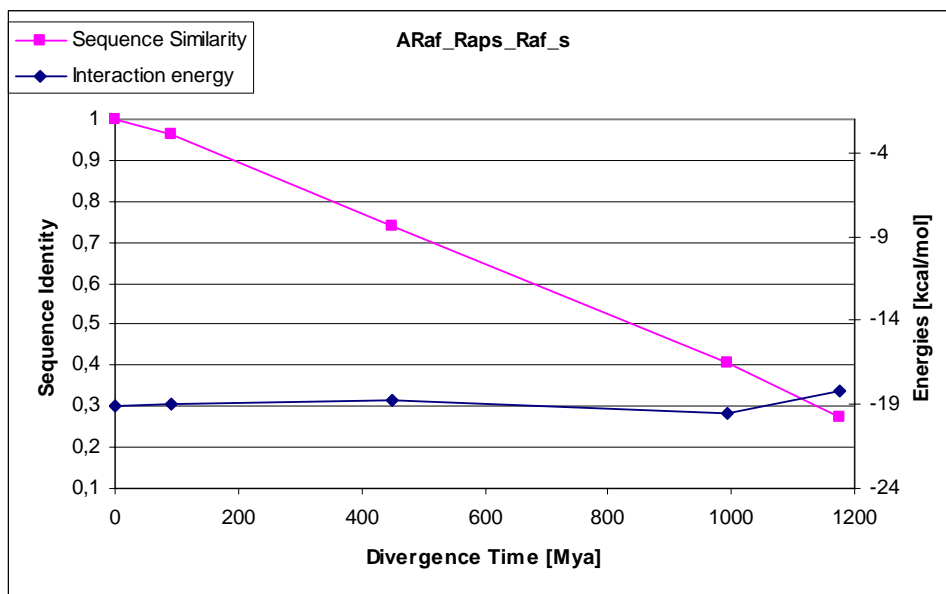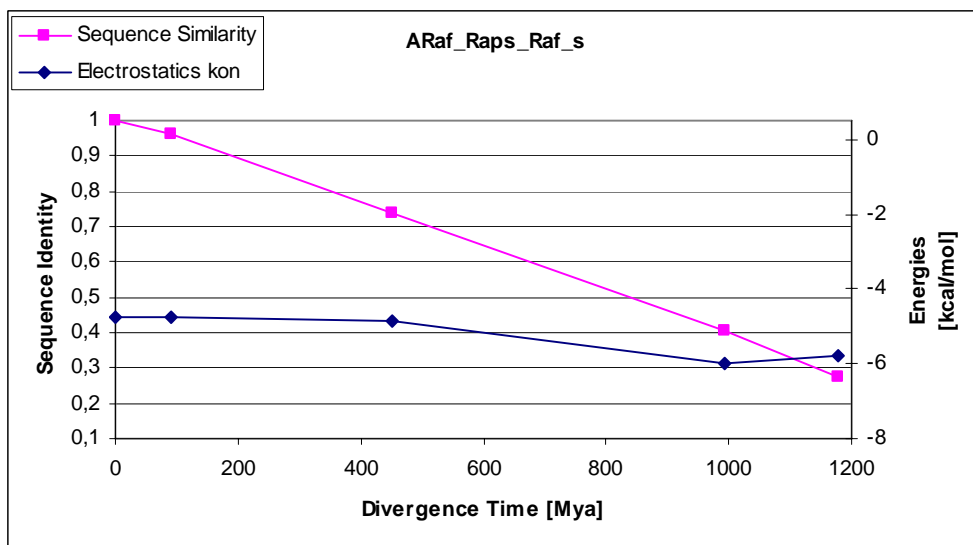

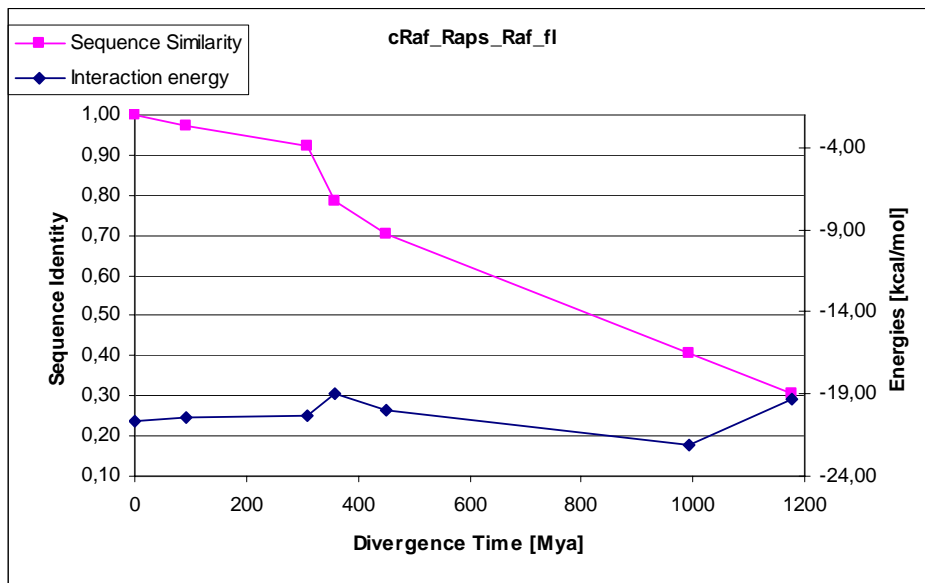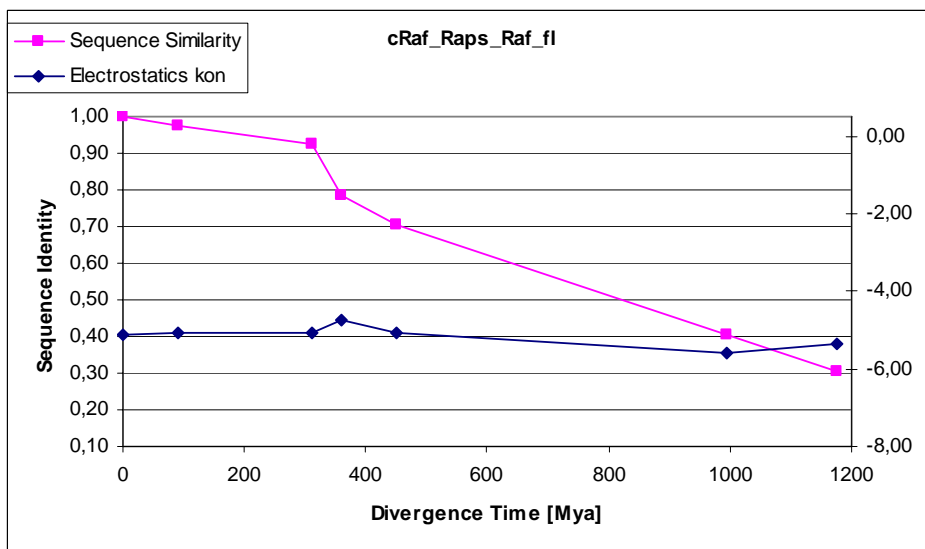

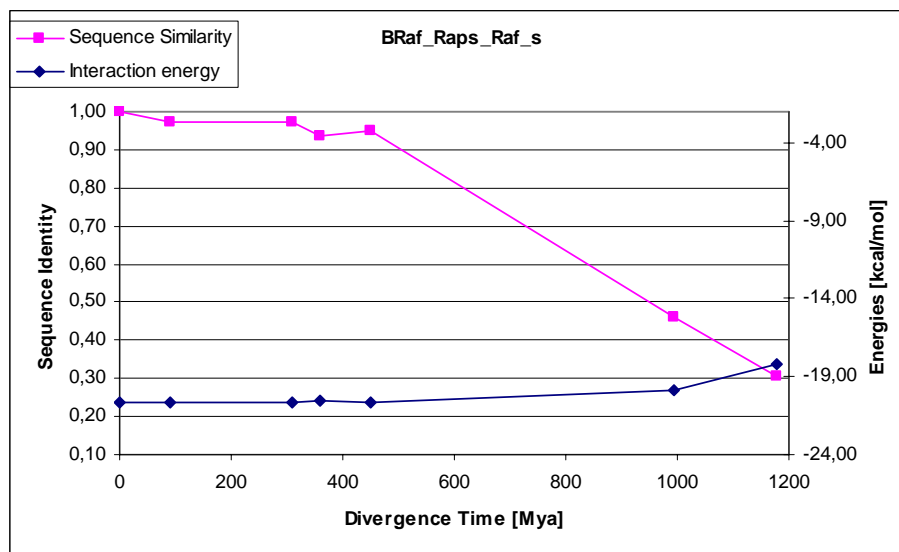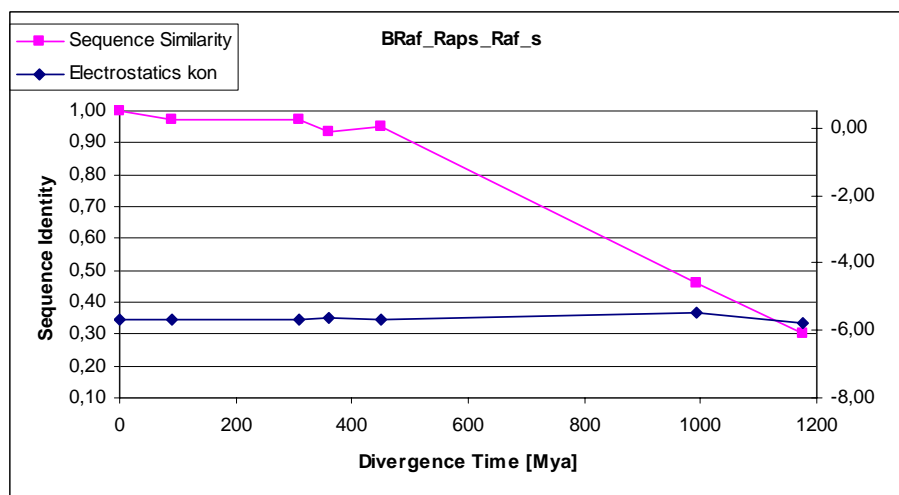

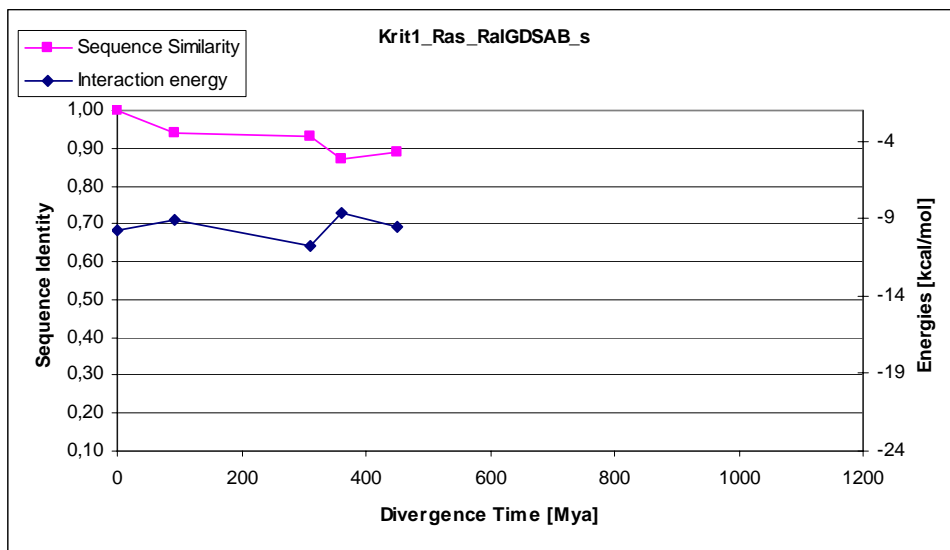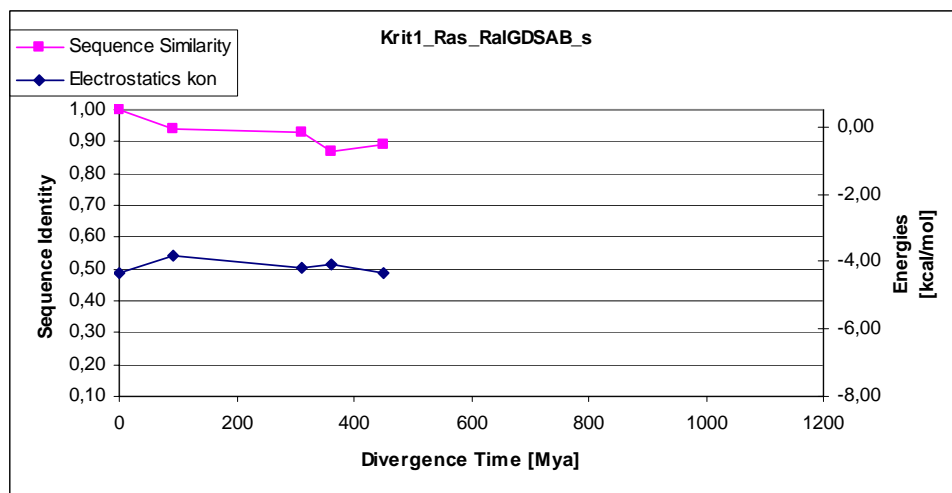

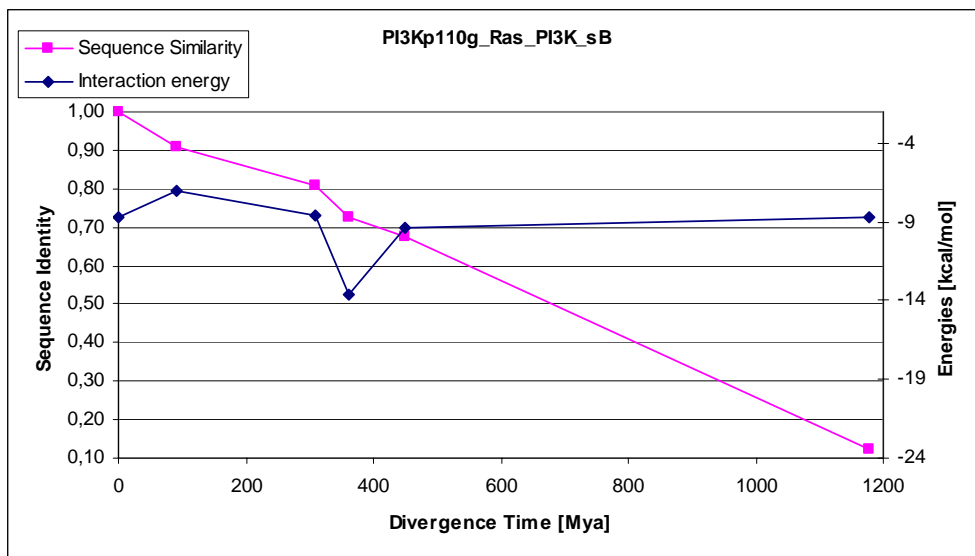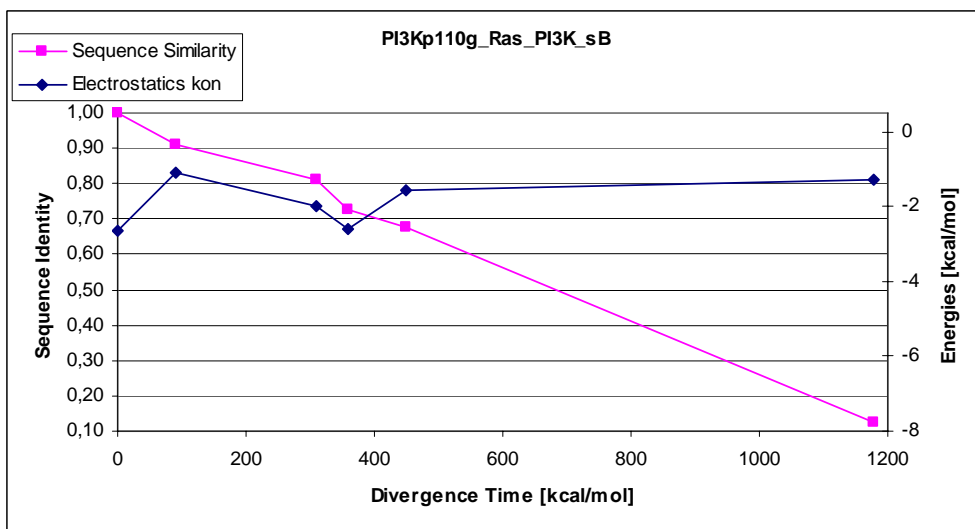

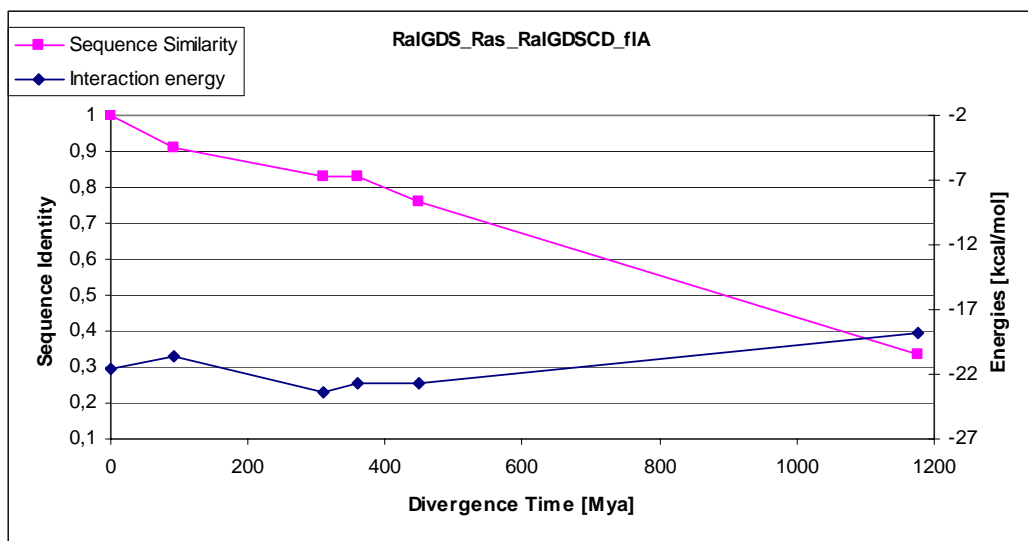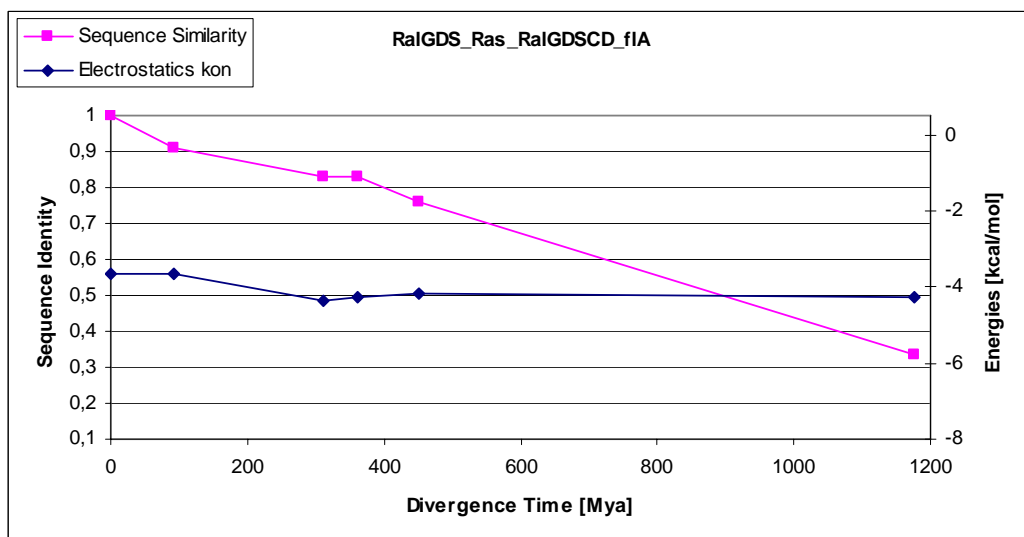

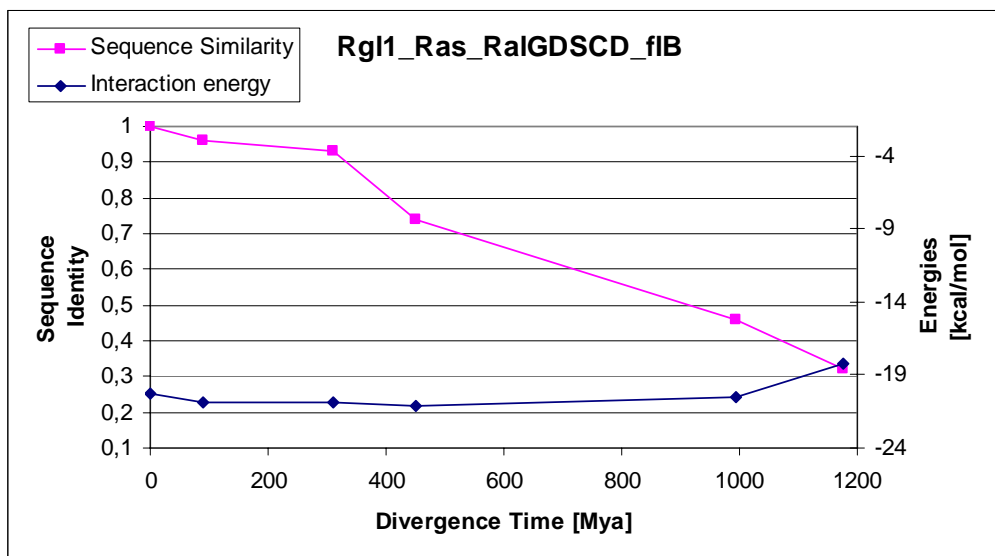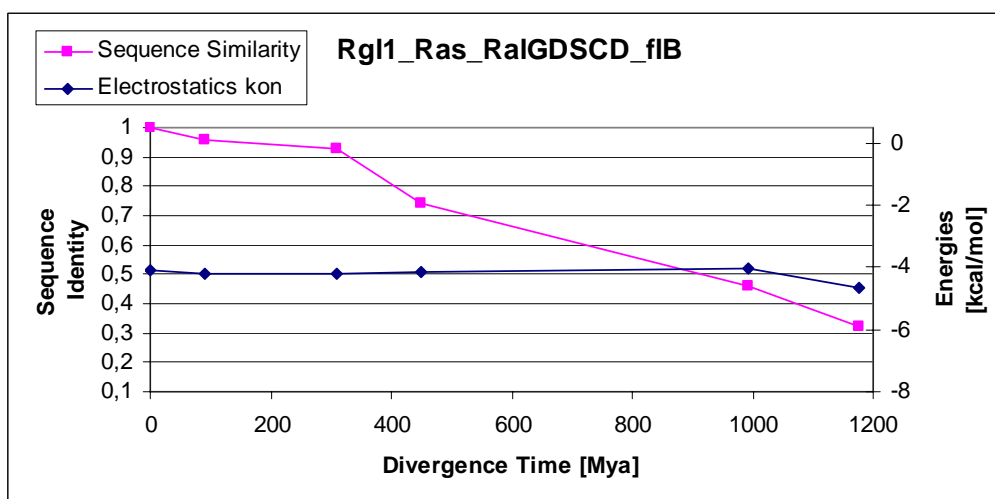

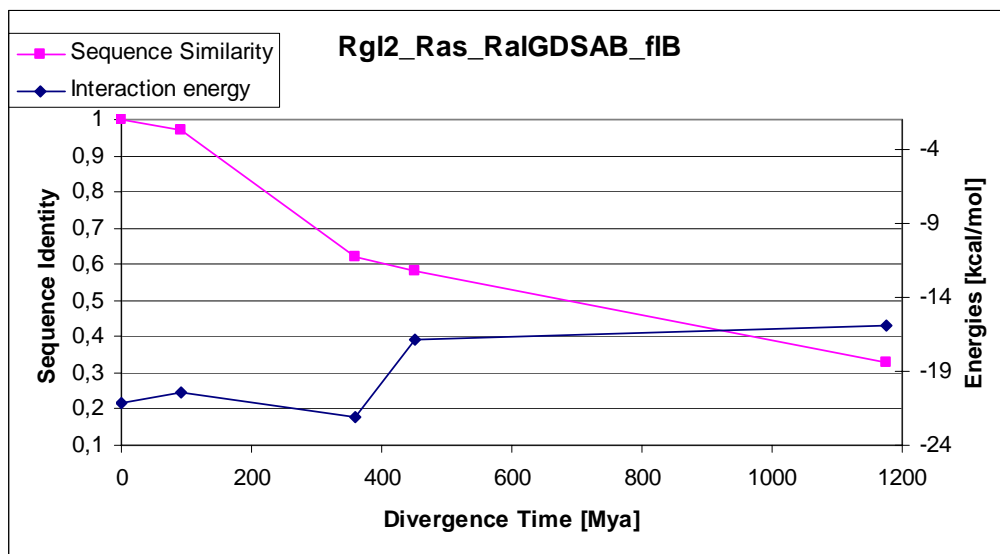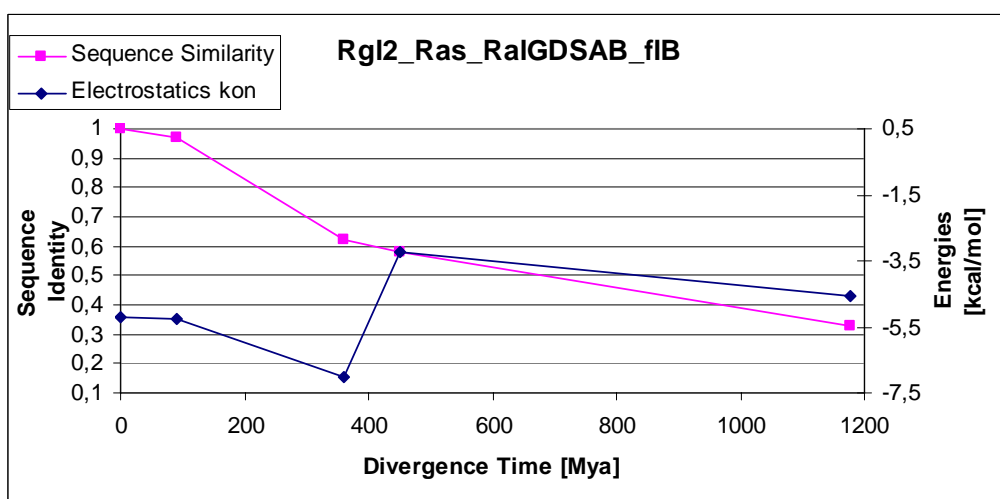

Supplement: Figure S3 — Diagrams of DGint and DGkon FoldX values plotted against the divergence time (0.07 MB PDF) [file pcbi.1000245.s003.pdf]

(A)

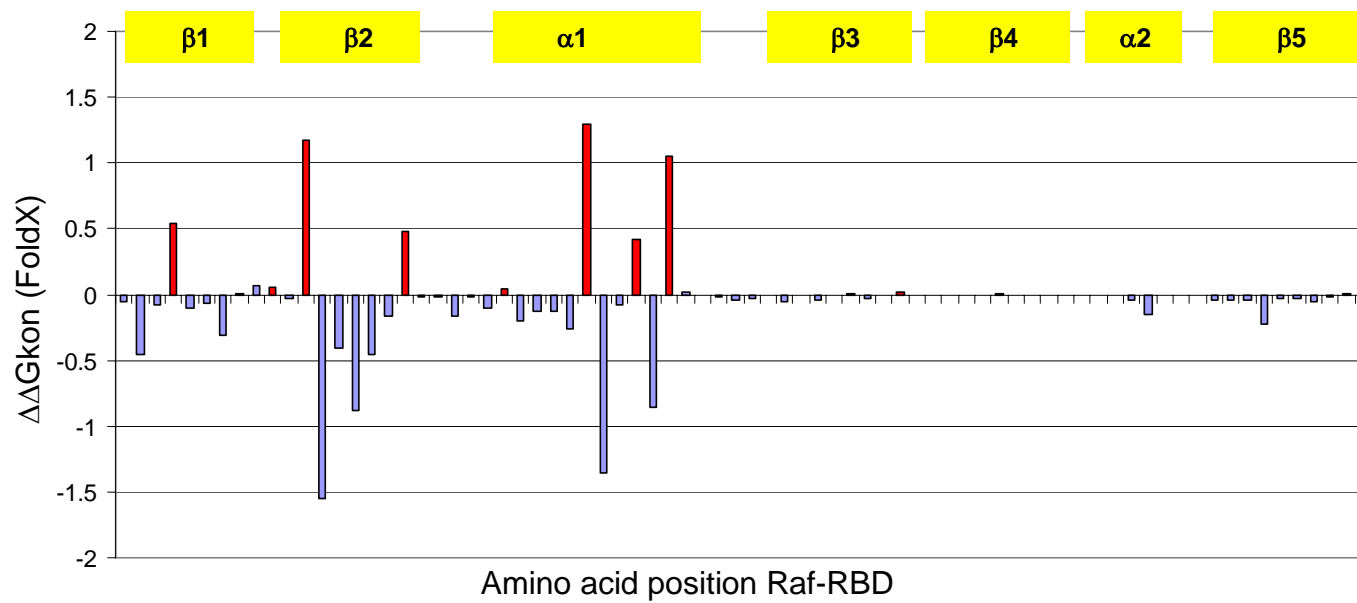

(B)

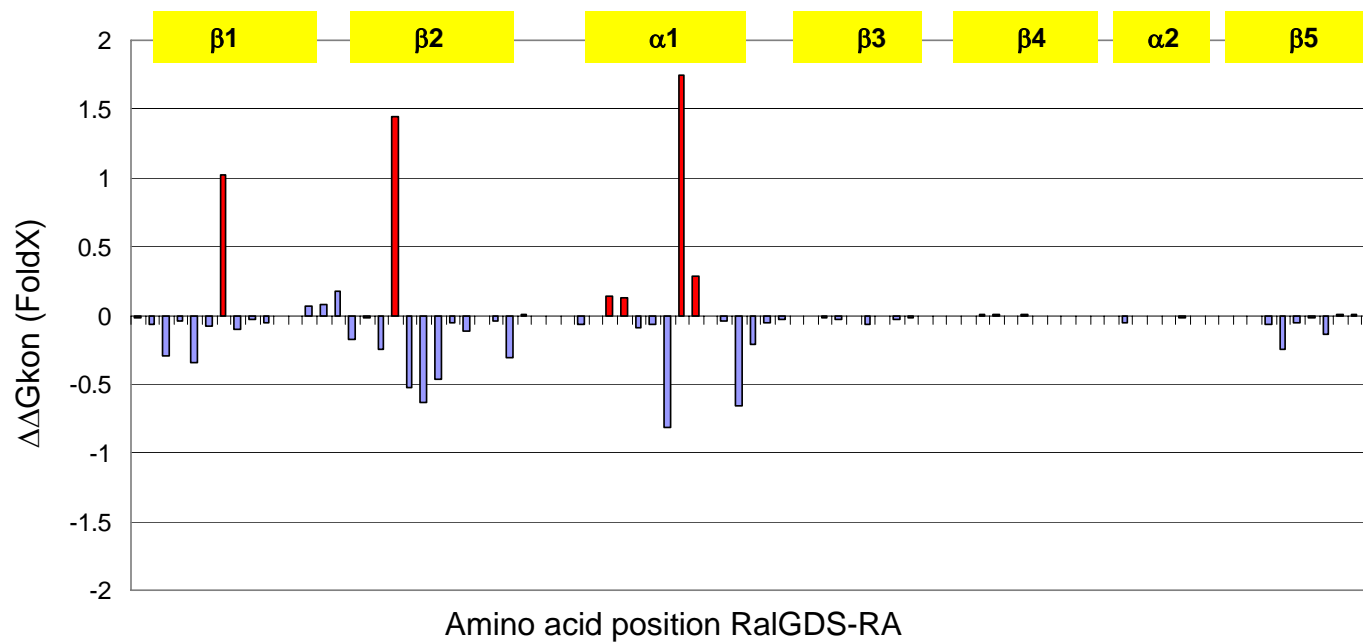

Supplement: Figure S4 — FoldX mutational scanning of RafRBD and RalGDS-RA. (A) Ras-RafRBD (pdb-entry 1GUA). (B) Ras-RalGDS (pdb entry 1LFD). Effect of all residues in Raf-RBD or RalGDS-RA on the contribution of DG kon as calculated by FoldX. Either positively charged residues were mutated to alanine (red) or negatively charged or neutral residues were mutated to lysine using FoldX and the difference compared to the WT DGkon was calculated and plotted for every amino acid position. (0.03 MB PDF) [file pcbi.1000245.s004.pdf]
