# Supplementary material for: Association Rate Constants of Ras-Effector Interactions Are Evolutionarily Conserved
Source: PLoS Comput Biol. 2008 Dec 19;4(12):e1000245. doi: 10.1371/journal.pcbi.1000245 (PMC2588540; doi:10.1371/journal.pcbi.1000245)
Supplement: Table S1 — Experimental and calculated association rate constants (0.03 MB PDF) [file pcbi.1000245.s005.pdf]

Table S1

(a) Ras-Raf at different salt concentrations

Data were taken from phD thesis from Kiel, C. (2003). Investigation of Ras-effector complexes with changed electrostatic properties. PhD thesis, Ruhr-Universitaet Bochum, Germany.

T=298 k

NaCl	kon exp (μM-1 s-1)	log kon exp	DG kon calc (FoldX)
0	60	1.77815125	-5.69
100	24	1.380211242	-3.37
200	17.3	1.238046103	-2.64
500	9.5	0.977723605	-1.82
800	7.4	0.86923172	-1.5

(b) Ras-RalGDS mutants

Data were taken from Kiel, C., Selzer, T., Shaul, Y., Schreiber, G., and Herrmann, C. (2004). PNAS, 101, 9223-9228.

Ras-RalGDS complex (wt and mutants)	kon exp (μM-1 s-1)	log kon exp	DG kon calc (FoldX)
R20R_RasK31E_RalGDS_CD	7.7	8.86E-01	-3.09
R20A_RasK31E_RalGDS_CD	2.5	3.98E-01	-2.68
S22K_RasK31E_RalGDS_CD	1.60E+01	1.204119983	-3.16
L23K_RasK31E_RalGDS_CD	8.9	9.49E-01	-3.22
N27K_RasK31E_RalGDS_CD	1.17E+01	1.068185862	-3.1
M30K_RasK31E_RalGDS_CD	1.62E+01	1.209515015	-3.38
K32A_RasK31E_RalGDS_CD	2.2	3.42E-01	-1.7
K48A_RasK31E_RalGDS_CD	5.6	7.48E-01	-3.01
D51A_RasK31E_RalGDS_CD	1.75E+01	1.243038049	-3.52
D51K_RasK31E_RalGDS_CD	3.03E+01	1.481442629	-3.94
K52A_RasK31E_RalGDS_CD	2	3.01E-01	-1.41
N54K_RasK31E_RalGDS_CD	1.58E+01	1.198657087	-3.58
L55K_RasK31E_RalGDS_CD	1.51E+01	1.178976947	-3.18
D56K_RasK31E_RalGDS_CD	1.85E+01	1.267171728	-3.83
E57A_RasK31E_RalGDS_CD	1.63E+01	1.212187604	-3.21
E57K_RasK31E_RalGDS_CD	1.91E+01	1.281033367	-3.31
D58K_RasK31E_RalGDS_CD	1.52E+01	1.181843588	-3.2
N92K_RasK31E_RalGDS_CD	2.14E+01	1.330413773	-3.19
Y93K_RasK31E_RalGDS_CD	2.17E+01	1.336459734	-3.19
D94K_RasK31E_RalGDS_CD	2.79E+01	1.445604203	-3.42
D58K_M30K_RasK31E_RalGDS_CD	1.73E+01	1.238046103	-3.45
D94K_M30K_RasK31E_RalGDS_CD	4.75E+01	1.67669361	-3.64
D58K_D51K_M30K_RasK31E_RalGDS_CD	1.10E+02	2.041392685	-4.27
D94K_D58K_D51K_M30K_RasK31E_RalGDS_CD	9.63E+01	1.983626287	-4.52
E57K_D56K_D51K_RasK31E_RalGDS_CD	4.30E+01	1.633468456	-4.68
D94K_D58K_RasK31E_RalGDS_CD	2.84E+01	1.45331834	-3.49
