# Supplementary material for: Association Rate Constants of Ras-Effector Interactions Are Evolutionarily Conserved
Source: PLoS Comput Biol. 2008 Dec 19;4(12):e1000245. doi: 10.1371/journal.pcbi.1000245 (PMC2588540; doi:10.1371/journal.pcbi.1000245)
Supplement: Table S2 — Divergence times and sequence identities for Ub domains (0.02 MB PDF) [file pcbi.1000245.s006.pdf]

Table S2 - page 1

Protein domain	organism	divergence t	Sequence Identity
AF6_RA1			
hsAF6_RA1	hs	0	1
mmAF6_RA1	mm	91	0.948453608
ggAF6_RA1	gg	310	0.958762887
xtAF6_RA1	xt	360	0.927835052
frAF6_RA1	fr	450	0.927835052
drAF6_RA1	dr	450	0.927835052
dmAF6_RA1	dm	993	0.721649485
dpAF6_RA1	dp	993	0.721649485
agAF6_RA1	ag	993	0.701030928
ceAF6_RA1	ce	1177	0.608247423
cbAF6_RA1	cb	1177	0.618556701
AF6_RA2			
hsAF6_RA2	hs	0	1
mmAF6_RA2	mm	91	0.943925234
ggAF6_RA2	gg	310	0.934579439
xtAF6_RA2	xt	360	0.831775701
frAF6_RA2	fr	450	0.710280374
drAF6_RA2	dr	450	0.747663551
dmAF6_RA2	dm	993	0.457943925
dpAF6_RA2	dp	993	0.457943925
agAF6_RA2	ag	993	0.439252336
ceAF6_RA2	ce	1177	0.271028037
cbAF6_RA2	cb	1177	0.261682243
ARaf			
hsARaf	hs	0	1.00
mmARaf	mm	91	0.96
frARaf	fr	450	0.73
drARaf	dr	450	0.75
dmRaf	dm	993	0.43
dpRaf	dp	993	0.43
agRaf	ag	993	0.41
ceRaf	ce	1177	0.28
cbRaf	cb	1177	0.27
CRaf			
hscRaf	hs	0	1.00
mmcRaf	mm	91	0.97
ggcRaf	gg	310	0.92
xtcRaf	xt	360	0.78
frcRaf	fr	450	0.71
drcRaf	dr	450	0.70
dmRaf	dm	993	0.41
dpRaf	dp	993	0.42
agRaf	ag	993	0.41
ceRaf	ce	1177	0.32
cbRaf	cb	1177	0.29
BRaf			
hsBRaf	hs	0	1.00
mmBRaf	mm	91	0.97
ggBRaf	gg	310	0.97
xtBRaf	xt	360	0.94
drBRaf	dr	450	0.95
dmRaf	dm	993	0.46
dpRaf	dp	993	0.46
agRaf	ag	993	0.46
amBRaf	am	993	0.47
ceRaf	ce	1177	0.32
cbRaf	cb	1177	0.29
Krit1			
hsKrit1	hs	0	1.00
mmKrit1	mm	91	0.94
ggKrit1	gg	310	0.93
xtKrit1	xt	360	0.87
frKrit1	fr	450	0.89

Table S2 - page 2

PI3K_p110-g

hsPI3Kp110g	hs	0	1.00
mmPI3Kp110g	mm	91	0.91
ggPI3Kp110g	gg	310	0.81
xtPI3Kp110g	xt	360	0.73
frPI3Kp110g	fr	450	0.67
drPI3Kp110g	dr	450	0.68
cePI3Kp110	ce	1177	0.17
cbPI3Kp110	cb	1177	0.14

RalGDS

hsRalGDS	hs	0	1
mmRalGDS	mm	91	0.91
ggRalGDS	gg	310	0.83
xtRalGDS	xt	360	0.83
frRalGDS	fr	450	0.84
drRalGDS	dr	450	0.68
ceRalGDS	ce	1177	0.33
cbRalGDS	cb	1177	0.34

Rgl1

hsRgl1	hs	0	1
mmRgl1	mm	91	0.96
ggRgl1	gg	310	0.93
frRgl1	fr	450	0.74
drRgl1	dr	450	0.74
dmRgl1	dm	993	0.47
dpRgl1	dp	993	0.47
agRgl1	ag	993	0.47
amRgl1	am	993	0.42
ceRalGDS	ce	1177	0.33

Rgl2

hsRgl2	hs	0	1
mmRgl2	mm	91	0.97
xtRgl2	xt	360	0.58
frRgl2	fr	450	0.62
ceRalGDS	ce	1177	0.33
