# Supplementary material for: Association Rate Constants of Ras-Effector Interactions Are Evolutionarily Conserved
Source: PLoS Comput Biol. 2008 Dec 19;4(12):e1000245. doi: 10.1371/journal.pcbi.1000245 (PMC2588540; doi:10.1371/journal.pcbi.1000245)
Supplement: Table S3 — Template structures used for homology modelling (0.01 MB PDF) [file pcbi.1000245.s007.pdf]

Table S3. Template structures used for homology modeling

Original pdb	Name of template	residues in Ras binding domain
1lfd	Ras_RalGDSAB_flB	Chain A: C17-D24; N29-D56; P60-I69; E71-I77; E79-S89; N92-R100
	Ras_RalGDSAB_S	Chain A: I18-L23; N29-T37; P43-D56
	Ras_RalGDS_ProS	Chain A: I18-S22; N29-T37; P43-D56
1lfd	Ras_RalGDSCD_flA	Chain C: D15-E57; E59-I69; E71-R100
	Ras_RalGDSCD_flB	Chain C: C17-D24; N29-D56; P60-I69; E71-I77; E79-S89; N92-R100
	Ras_RalGDSCD_S	Chain C: I18-L23; N29-T37; P43-D56
	Ras_RalGDSCD_flD	Chain C: D15-L23; M30-I69; H73-L75; N80-M87; D94-K98
1he8	Ras_PI3K_SB	Chain A: F221-R226; T228-V235; G242-A253
1gua	Raps_Raf_fl	Chain B: T57-P63; K65-H103; K109-I122; E124-D129
	Raps_Raf_S	Chain B: T57-L62; K65-G75; H79-L91
