# Supplementary material for: Association Rate Constants of Ras-Effector Interactions Are Evolutionarily Conserved
Source: PLoS Comput Biol. 2008 Dec 19;4(12):e1000245. doi: 10.1371/journal.pcbi.1000245 (PMC2588540; doi:10.1371/journal.pcbi.1000245)
Supplement: Table S4 — FoldX results for all homology models (0.01 MB PDF) [file pcbi.1000245.s008.pdf]

Table _S4

Ras-AF6_RA1

		Species	Interaction energy	Electrostatics	Electrostatics kon	Sequence Similarity
147	AF6RA1_Ras_RalGDSAB_flB	hs	-15.18	-8.9	-5.89	1
148	AF6RA1_Ras_RalGDSAB_flB	mm	-20.68	-12.97	-6.99	0.948453608
149	AF6RA1_Ras_RalGDSAB_flB	gg	-19.08	-11.73	-6.62	0.958762887
152	AF6RA1_Ras_RalGDSAB_flB	xt	-18.19	-10.84	-6.03	0.927835052
150	AF6RA1_Ras_RalGDSAB_flB	fr	-19.47	-12.22	-6.85	0.927835052
151	AF6RA1_Ras_RalGDSAB_flB	dr	-17.09	-10.7	-5.99	0.927835052
153	AF6RA1_Ras_RalGDSAB_flB	dm	-18.42	-10.92	-5.82	0.721649485
154	AF6RA1_Ras_RalGDSAB_flB	dp	-18.42	-10.92	-5.82	0.721649485
155	AF6RA1_Ras_RalGDSAB_flB	ag	-21.13	-12.77	-7.08	0.701030928
156	AF6RA1_Ras_RalGDSAB_flB	ce	-17.48	-8.85	-4.65	0.608247423
157	AF6RA1_Ras_RalGDSAB_flB	cb	-17.51	-8.23	-4.27	0.618556701

Ras-AF6_RA2

		Species	Interaction energy	Electrostatics	Electrostatics kon	Sequence Similarity
457	AF6_RA2_Ras_RalGDS_ProS	hs	-10.35	-3.85	-2.87	1
458	AF6_RA2_Ras_RalGDS_ProS	mm	-10.28	-3.83	-2.85	0.943925234
459	AF6_RA2_Ras_RalGDS_ProS	gg	-11.32	-3.95	-2.84	0.934579439
462	AF6_RA2_Ras_RalGDS_ProS	xt	-10.64	-3.62	-2.66	0.831775701
460	AF6_RA2_Ras_RalGDS_ProS	fr	-9.53	-3.84	-3.03	0.710280374
461	AF6_RA2_Ras_RalGDS_ProS	dr	-10.35	-3.85	-2.87	0.747663551
463	AF6_RA2_Ras_RalGDS_ProS	dm	-8.73	-3.75	-2.84	0.457943925
464	AF6_RA2_Ras_RalGDS_ProS	dp	-8.73	-3.75	-2.84	0.457943925
465	AF6_RA2_Ras_RalGDS_ProS	ag	-11.79	-5.04	-2.65	0.439252336
466	AF6_RA2_Ras_RalGDS_ProS	ce	-9.38	-3.09	-2.35	0.271028037
467	AF6_RA2_Ras_RalGDS_ProS	cb	-8.39	-2.86	-2.44	0.261682243

Ras-ARaf

		Species	Interaction energy	Electrostatics	Electrostatics kon	Sequence Similarity
718	ARaf_Raps_Raf_s	hs	-19.06	-6.5	-4.78	1
721	ARaf_Raps_Raf_s	mm	-19.02	-6.47	-4.75	0.962025316
726	ARaf_Raps_Raf_s	fr	-18.88	-6.5	-4.84	0.734177215
728	ARaf_Raps_Raf_s	dr	-18.72	-6.59	-4.92	0.746835443
735	Raf_Raps_Raf_s	ag	-19.48	-9.05	-5.99	0.405063291
737	Raf_Raps_Raf_s	ce	-18.14	-6.98	-5.79	0.278481013
738	Raf_Raps_Raf_s	cb	-18.14	-6.98	-5.79	0.265822785

Ras-CRaf

		Species	Interaction energy	Electrostatics	Electrostatics kon	Sequence Similarity
768	cRaf_Raps_Raf_fl	hs	-20.63	-6.74	-5.11	1.00
771	cRaf_Raps_Raf_fl	mm	-20.48	-6.69	-5.07	0.97

774	cRaf_Raps_Raf_fl	gg	-20.32	-6.69	-5.06	0.92
781	cRaf_Raps_Raf_fl	xt	-18.95	-6.35	-4.76	0.78
776	cRaf_Raps_Raf_fl	fr	-19.36	-6.61	-5.09	0.71
778	cRaf_Raps_Raf_fl	dr	-20.67	-6.74	-5.07	0.70
786	Raf_Raps_Raf_fl	ag	-22.11	-7.79	-5.58	0.41
788	Raf_Raps_Raf_fl	ce	-19.67	-7.19	-5.32	0.32
789	Raf_Raps_Raf_fl	cb	-18.87	-7.15	-5.42	0.29

Ras-BRaf

		Species	Interaction energy	Electrostatics	Electrostatics kon	Sequence Similarity
719	BRaf_Raps_Raf_s	hs	-20.61	-8.61	-5.66	1.00
722	BRaf_Raps_Raf_s	mm	-20.61	-8.61	-5.66	0.97
724	BRaf_Raps_Raf_s	gg	-20.61	-8.61	-5.66	0.97
731	BRaf_Raps_Raf_s	xt	-20.49	-8.57	-5.61	0.94
729	BRaf_Raps_Raf_s	dr	-20.61	-8.61	-5.66	0.95
735	Raf_Raps_Raf_s	ag	-19.48	-9.05	-5.99	0.46
736	BRaf_Raps_Raf_s	am	-20.23	-6.90	-4.99	0.47
737	Raf_Raps_Raf_s	ce	-18.14	-6.98	-5.79	0.32
738	Raf_Raps_Raf_s	cb	-18.14	-6.98	-5.79	0.29

Ras-Krit1

		Species	Interaction energy	Electrostatics	Electrostatics kon	Sequence Similarity
344	Krit1_Ras_RalGDSAB_s	hs	-9.74	-6.83	-4.34	1.00
345	Krit1_Ras_RalGDSAB_s	mm	-9.07	-6.73	-3.85	0.94
346	Krit1_Ras_RalGDSAB_s	gg	-10.73	-7.13	-4.19	0.93
348	Krit1_Ras_RalGDSAB_s	xt	-8.66	-6.40	-4.10	0.87
347	Krit1_Ras_RalGDSAB_s	fr	-9.57	-6.78	-4.36	0.89

Ras-PI3Kp110gamma

		Species	Interaction energy	Electrostatics	Electrostatics kon	Sequence Similarity
672	PI3Kp110g_Ras_PI3K_sB	hs	-8.7	-2.9	-2.64	1.00
676	PI3Kp110g_Ras_PI3K_sB	mm	-7.08	-0.79	-1.1	0.91
680	PI3Kp110g_Ras_PI3K_sB	gg	-8.55	-2.35	-1.98	0.81
690	PI3Kp110g_Ras_PI3K_sB	xt	-13.56	-3.43	-2.62	0.73
683	PI3Kp110g_Ras_PI3K_sB	fr	-8.96	-1.7	-1.59	0.67
687	PI3Kp110g_Ras_PI3K_sB	dr	-9.85	-1.54	-1.5	0.68
699	PI3Kp110_Ras_PI3K_sB	ce	-7.7	-0.35	-0.8	0.13
700	PI3Kp110_Ras_PI3K_sB	cb	-9.65	-3.26	-1.78	0.12

Ras-RalGDS

		Species	Interaction energy	Electrostatics	Electrostatics kon	Sequence Similarity
9	RalGDS_Ras_RalGDSCD_flA	hs	-21.53	-8.31	-3.65	1

10 RalGDS_Ras_RalGDSCD_flA	mm	-20.56	-8.39	-3.65	0.91
11 RalGDS_Ras_RalGDSCD_flA	gg	-23.35	-8.86	-4.36	0.83
14 RalGDS_Ras_RalGDSCD_flA	xt	-22.7	-8.68	-4.25	0.83
12 RalGDS_Ras_RalGDSCD_flA	fr	-23.12	-8.87	-4.38	0.84
13 RalGDS_Ras_RalGDSCD_flA	dr	-22.16	-8.42	-3.98	0.68
15 RalGDS_Ras_RalGDSCD_flA	ce	-18.54	-8.68	-4.23	0.33
16 RalGDS_Ras_RalGDSCD_flA	cb	-19.04	-8.73	-4.35	0.34

Ras-Rgl1

	Species	Interaction energy	Electrostatics	Electrostatics kon	Sequence Similarity
40 Rgl1_Ras_RalGDSCD_flB	hs	-20.32	-8.63	-4.11	1
43 Rgl1_Ras_RalGDSCD_flB	mm	-20.93	-8.68	-4.18	0.96
46 Rgl1_Ras_RalGDSCD_flB	gg	-20.93	-8.68	-4.18	0.93
48 Rgl1_Ras_RalGDSCD_flB	fr	-21.1	-8.33	-4.17	0.74
51 Rgl1_Ras_RalGDSCD_flB	dr	-21.02	-8.59	-4.13	0.74
55 Rgl1_Ras_RalGDSCD_flB	dm	-20.46	-8.06	-4.07	0.47
56 Rgl1_Ras_RalGDSCD_flB	dp	-20.46	-8.06	-4.07	0.47
57 Rgl1_Ras_RalGDSCD_flB	ag	-19.62	-7.94	-3.89	0.47
58 Rgl1_Ras_RalGDSCD_flB	am	-21.5	-8.48	-4.12	0.42
59 RalGDS_Ras_RalGDSCD_flB	ce	-18.23	-9.11	-4.63	0.32

Ras-Rgl2

	Species	Interaction energy	Electrostatics	Electrostatics kon	Sequence Similarity
19 Rgl2_Ras_RalGDSAB_flB	hs	-21.13	-9.66	-5.21	1
22 Rgl2_Ras_RalGDSAB_flB	mm	-20.45	-9.44	-5.28	0.97
31 Rgl2_Ras_RalGDSAB_flB	xt	-22.15	-11.27	-7	0.62
27 Rgl2_Ras_RalGDSAB_flB	fr	-16.92	-5.83	-3.24	0.58
37 RalGDS_Ras_RalGDSAB_flB	ce	-15.9	-8.36	-4.55	0.33
