# Supplementary material for: Association Rate Constants of Ras-Effector Interactions Are Evolutionarily Conserved
Source: PLoS Comput Biol. 2008 Dec 19;4(12):e1000245. doi: 10.1371/journal.pcbi.1000245 (PMC2588540; doi:10.1371/journal.pcbi.1000245)
Supplement: Table S5 — Mean values and STDEV for homology models for ortholog complexes (0.01 MB PDF) [file pcbi.1000245.s009.pdf]

Table S5

	Interaction energy (kcal/mol)		Electrostatics kon (kcal/mol)	
	Mean	StDev	Mean	StDev
AF6_RA1_Ras_RalGDSAB_flB	-18.31833333	1.71816682	-6.092857143	0.808573111
AF6_RA2_Ras_RalGDS_ProS	-10.16642857	0.760299909	-2.763095238	0.185353587
ARaf_Raps_Raf_s	-18.9	0.490917508	-5.238	0.60130691
cRaf_Raps_Raf_fl	-20.25357143	1.030156599	-5.147142857	0.260237986
BRaf_Raps_Raf_s	-20.13214286	0.920768959	-5.647142857	0.088640526
Krit1_Ras_RalGDSAB_s	-9.554	0.782834593	-4.168	0.207773916
PI3Kp110g_Ras_PI3K_sB	-9.328333333	2.208860943	-1.8625	0.663639586
RalGDS_Ras_RalGDSCD_flA	-21.595	1.69331332	-4.063333333	0.325433045
Rgl1_Ras_RalGDSCD_flB	-20.33	1.067276909	-4.214583333	0.21044249
Rgl2_Ras_RalGDSAB_flB	-19.31	2.739425122	-5.056	1.360782863
