# Supplementary material for: Association Rate Constants of Ras-Effector Interactions Are Evolutionarily Conserved
Source: PLoS Comput Biol. 2008 Dec 19;4(12):e1000245. doi: 10.1371/journal.pcbi.1000245 (PMC2588540; doi:10.1371/journal.pcbi.1000245)
Supplement: Table S6 — Modelling parameters (0.05 MB PDF) [file pcbi.1000245.s010.pdf]

Table S6

Reaction Nr.	Reaction	kon (M-1 s-1)	koff (s-1)	k forw (s-1)	remarks
1	A + GEF ↔ A_GEF	10e6	0.6		estimation
2	A_GEF → GEF_act			1	estimation
3	GEF_act + RasD ↔ GEF_act_RasD	15e6	1.3		Schoeberl, 2002
4	GEF_act_RasD → GEF_act + RasT			0.1434	Yamada, 2004
5	RasT + Raf ↔ RasT_Raf	29.6e6	5.7		Kiel, 2003
6	RasT_Raf → RasT + Raf_act			0.7624	Yamada, 2004
8	Raf_act → X			1	estimation
9	RasT + GAP ↔ RasT_GAP	2.854e6	0.96		Yamada, 2004
10	RasT_GAP → RasD + GAP			7.76	Yamada, 2004
11	X_act → X_deg			0.001	estimation

Kiel C (2003) Investigation of Ras-effector complexes with changed electrostatic properties. PhD thesis, Ruhr-Universitaet Bochum, Germany.
Schoeberl B, Eichler-Jonsson C, Gilles ED, Mueller G (2002) Computational modeling of the dynamics of the MAP kinase cascade activated by surface and internalized EGF receptors. Nat Biotech 20: 370-375.
Yamada S, Taketomi T, Yoshimura A (2004) Model analysis of difference between EGF pathway and FGF pathway. Biochem Biophys Res Commun 314: 1113-1120.
